# Transcriptomic and metabolomic data reveal key genes that are involved in the phenylpropanoid pathway and regulate the floral fragrance of *Rhododendron fortunei*

**DOI:** 10.1186/s12870-022-04016-7

**Published:** 2023-01-05

**Authors:** Guoxia Yang, Yi Qin, Yonghong Jia, Xiaohong Xie, Dongbin Li, Baoxin Jiang, Qu Wang, Siyu Feng, Yueyan Wu

**Affiliations:** 1 College of Biological and Environmental Sciences, Zhejiang Wanli University, Ningbo, 315100 Zhejiang China; 2 Ningbo Forest Farm, Ningbo, 315100 Zhejiang China

**Keywords:** *Rhododendron*, Floral aroma, Benzenes/phenylpropanoids, Metabolome and transcriptome analyses, Phenylpropanoid biosynthesis pathway, Salicylic acid carboxyl methyl transferase

## Abstract

**Background:**

To reveal the key genes involved in the phenylpropanoid pathway, which ultimately governs the fragrance of *Rhododendron fortunei*, we performed a comprehensive transcriptome and metabolomic analysis of the petals of two different varieties of two alpine rhododendrons: the scented *R. fortunei* and the unscented *Rhododendron* ‘Nova Zembla’.

**Results:**

Our transcriptomic and qRT–PCR data showed that nine candidate genes were highly expressed in *R. fortunei* but were downregulated in *Rhododendron* ‘Nova Zembla’. Among these genes, *EGS* expression was significantly positively correlated with various volatile benzene/phenylpropanoid compounds and significantly negatively correlated with the contents of various nonvolatile compounds, whereas *CCoAOMT*, *PAL*, *C4H,* and *BALDH* expression was significantly negatively correlated with the contents of various volatile benzene/phenylpropanoid compounds and significantly positively correlated with the contents of various nonvolatile compounds. *CCR*, *CAD*, *4CL,* and *SAMT* expression was significantly negatively correlated with the contents of various benzene/phenylpropanoid compounds. The validation of *RfSAMT* showed that the *RfSAMT* gene regulates the synthesis of aromatic metabolites in *R. fortunei*.

**Conclusion:**

The findings of this study indicated that key candidate genes and metabolites involved in the phenylpropanoid biosynthesis pathway may govern the fragrance of *R. fortunei*. This lays a foundation for further research on the molecular mechanism underlying fragrance in the genus *Rhododendron*.

**Supplementary Information:**

The online version contains supplementary material available at 10.1186/s12870-022-04016-7.

## Background

*Rhododendron* is the largest genus in the heath family (Ericaceae) and the largest genus of woody angiosperms found in China. Members of this genus are widely distributed throughout the Northern Hemisphere, from tropical Southeast Asia to northeastern Australia [1]. Rhododendrons are world-famous ornamental flowers widely used in landscaping and indoor beautification. There are approximately 1200 species of rhododendrons, of which more than 540 are endemic to southern and southwestern China [1, 2]. Taxonomically, the *Rhododendron* genus is complex, with varieties grouped into scented and unscented types depending on their aroma. *Rhododendron fortunei* is a scented rhododendron in the subgenus *Azaleastrum* [3] and has large, pink, and fragrant flowers; it is also native to China [4]. *Rhododendron fortunei* is widely distributed along sunny ridges or as a secondary layer in forests at an altitude of 620–2000 m above sea level [5, 6], and it has high ornamental value in gardens. *Rhododendron* ‘Nova Zembla’ is a hybrid in the subgenus *Azaleastrum*. It is evergreen, and its flowers are broad and funnel-shaped, with dark rose-red petals and dark maroon spots present on the uppermost petals. *Rhododendron* ‘Nova Zembla’ flowers are unscented. This variety can tolerate a low temperature of − 28 °C and can survive in high-altitude areas, and it is one of the best-selling varieties of alpine rhododendrons. Compared with research on other flower characteristics, such as flower colour and type, research on flower fragrance is lacking. Currently, research on synthesis pathways and key genes involved in plant floral aromatic metabolites has become a topic of interest [7].

Floral aroma is an important plant trait and plays a key role in plant growth, development, and evolution. Floral aroma has protective functions and attracts both pollinators and seed dispersers [8]. It is also an important indicator in the evaluation and assessment of ornamental plant quality. Thus, floral aroma is an economically valuable trait. Plant floral aroma is a manifestation of a series of low-molecular-weight volatile organic compounds (VOCs) that are produced by the interaction of plants with biotic and abiotic factors. VOCs pass through plant cell membranes and are ultimately released into the surrounding environment [9, 10]. To date, more than 1700 floral VOCs have been identified [11]. VOCs can be classified into five categories according to their biosynthetic sources, namely, terpenoids, benzene/phenylpropanoid compounds, fatty acid derivatives, amino acid derivatives, and a few other specific compounds [9]. In recent years, ornamental plant researchers and breeders have paid increasing amounts of attention to some relatively little-studied aromatic ornamental plants. The main VOCs in aromatic plant species such as rose, petunia, bearded iris, freesia, lily, anthurium, and *Barringtonia racemosa* have been detected and analysed. Shi et al. [12] summarized the components of rose floral, and they found that Chinese roses principally produce lipid-derived alcohols, esters and phenylpropanoids, whereas the major scent components of European roses are phenylpropanoids and a number of monoterpenes. The main aromatic compounds of the varieties Dianhong and Mohong were terpenes. Benzenes/phenylpropanoids are the main components of petunia fragrance, but also a small amount of terpenoids contribute to this aroma. Terpenes, alcohols and esters contribute the most to the fragrance of bearded iris [13, 14], and the main VOCs produced by freesia are terpenoids, especially monoterpenes [15]. Kong et al. [16] investigated the floral aroma of lily varieties with different fragrances and found that the aromatic compounds varied between different varieties, of which (E)-b-ocimene was the main aromatic compound. Wei et al. [17] detected VOCs in two varieties of Anthurium; their analysis revealed that the floral characteristics of Mystral were predominantly caused by terpenoids (70%), although there were smaller amounts of benzene/phenylpropanoid compounds present (28.5%). The main floral components of *Barringtonia racemosa* are terpenoids, mainly linalool, and benzene/phenylpropanoid compounds, mainly phenylacetaldehyde [18].

In recent years, high-throughput sequencing methods have been widely used to study the relationship between the contents of various secondary metabolites in plant organs and corresponding differentially expressed genes (DEGs) [19]. Metabolomics and transcriptomic association analysis enables analysis of coexpressed genes and metabolites and systematic analysis of molecular regulatory mechanisms [20]. By combining metabolome and transcriptome analyses, Ying et al. [19] analysed the key metabolites related to pulp colouring. They further constructed a regulatory network of cherry pulp colouring and elucidated its biosynthetic regulatory mechanisms. Zhang et al. [21] performed metabolomic and transcriptomic analyses to identify candidate genes involved in anthocyanin and proanthocyanidin abundance in young fruit of the pear cultivar Clapp Favorite and its red mutant cultivar Red Clapp Favorite. To identify the main genes and metabolites involved in the anthocyanin biosynthesis pathway in microrose and to understand the main genes and metabolites involved in the anthocyanin biosynthesis pathway in rosettes, Lu et al. [22] used A-type (wild-type (WT)) Neptune King and its natural mutant Queen as experimental materials to conduct a transcriptomic and metabolomic analysis. Compared with WT rose, the rose mutant presented a higher content of various flavonoids and altered gene expression patterns. Mei et al. [23] integrated metabolomic and transcriptomic data of petals of two tea plant varieties, white-flowered ZJ and pink-flowered BT, the results of which revealed a relationship between tea plant colour and aroma, that is, the relationship between anthocyanins and benzene/phenylpropanoid VOCs. By integrating data from transcriptomic and metabolomic analysis, Dhandapani et al. [24] found that there were 43 VOCs in *Magnolia*, of which 46.9% were terpenes, 38.9% were volatile esters, and 5.2% were benzenes/phenylpropanoids, all of which are related to floral compounds. Moreover, the expression levels of most of the individual synthesis-related genes were found to be higher in the flowers than in the leaves.

At present, research on the molecular mechanism underlying the synthesis of aromatic compounds in rhododendron is lacking, but this type of research is necessary for breeding new rhododendron varieties with aromatic flowers. Due to the limitations of GC–MS technology for the detection of nonvolatile metabolites, the precursors of floral aromatic compounds, such as p-hydroxycinnamic acid and methylglutaric acid, cannot be reasonably measured during the dynamic metabolic changes in organisms. However, by applying LC–MS positive (POS) and LC–MS negative (NEG) techniques, we can largely detect the accumulation of various nonvolatile metabolites. When the LC–MS POS and LC–MS NEG techniques were used, better separation was observed for compounds with different pKa values. In this study, a combined transcriptomic and metabolomic analysis was used to study differences in gene expression and metabolite accumulation in the petals of two different rhododendron varieties (a scented rhododendron and an unscented rhododendron). Focusing on the study of the differentially accumulated benzenes/phenylpropanoids of the two rhododendron varieties and the dynamic changes in benzenes/phenylpropanoids in *R. fortunei* at different developmental stages and screening out key candidate genes involved in the phenylpropanoid pathway, we elucidated the synthesis mechanism of benzene/phenylpropanoid floral aromatic compounds. We found that key genes involved in the phenylpropanoid pathway regulate the fragrance of *R. fortunei*, which lays a foundation for further research on the molecular mechanism of aromatic compound synthesis in rhododendron and provides a scientific basis for the breeding of novel scented rhododendron varieties.

## Results

### Multivariate statistical analysis of the metabolome

**Fig. 1:**
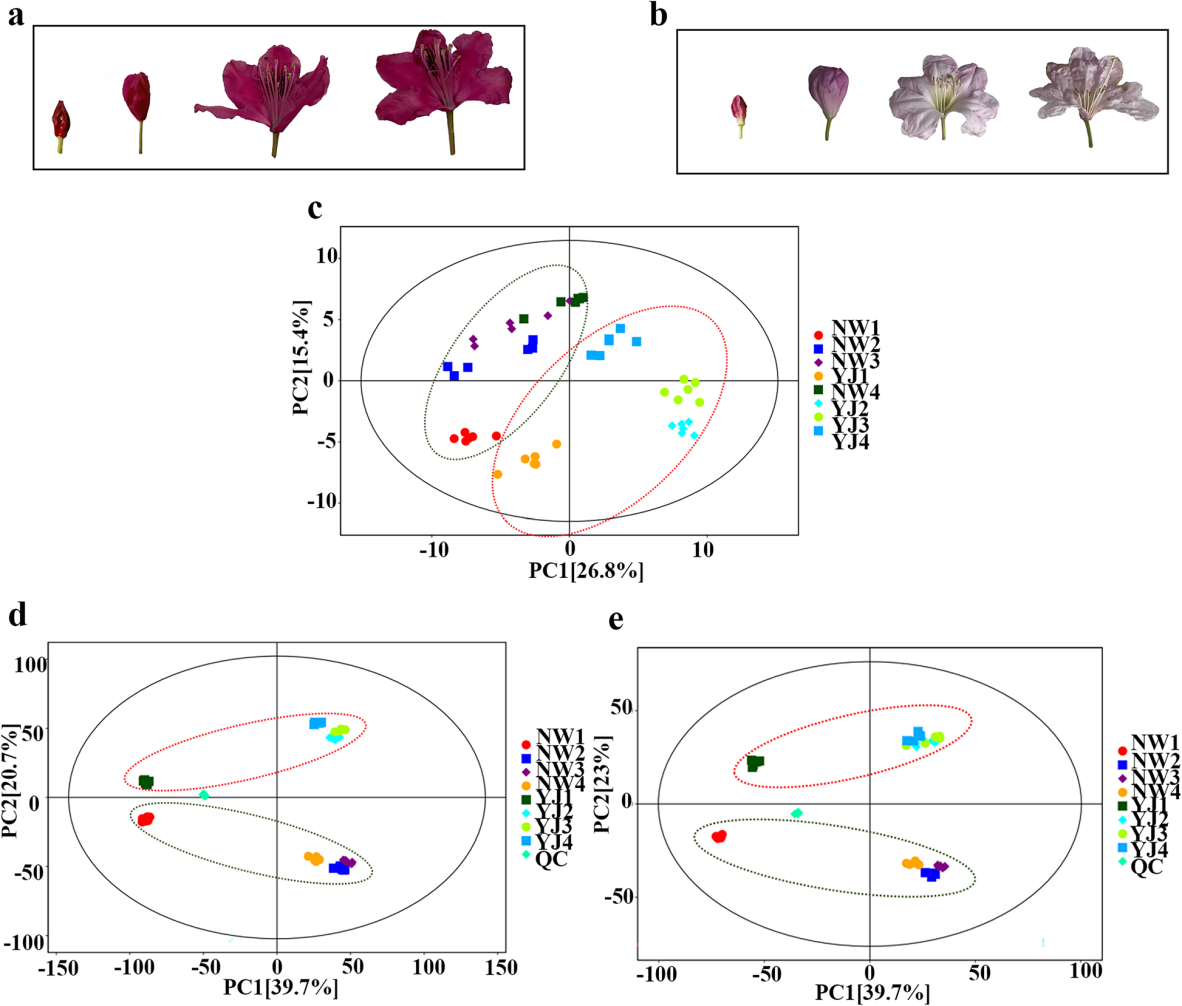
PCA results of metabolites in the petals of two rhododendron varieties (NW and YJ) at four developmental stages. **a** *Rhododendron* ‘Nova Zembla’ experimental material, **b** *R. fortunei* experimental material, **c** PCA results of metabolites detected by GC–TOF–MS, **d** PCA results of detected metabolites via LC–MS (POS), **e** PCA of metabolites detected via LC–MS (NEG)

In this study, GC–TOF–MS and LC–MS were used to detect metabolites in the petals of *Rhododendron* ‘Nova Zembla’ (NW) (Fig. 1a) and *R. fortunei* (YJ) (Fig. 1b) at different developmental stages, and nontargeted metabolomics methods were used to further analyse the differentially accumulated metabolites in the petals of the two rhododendron varieties and the dynamic changes in the accumulation of metabolites in the petals of YJ at four different developmental stages. The identified metabolites were divided into seven categories (Table 1), namely, benzene/phenylpropanoid compounds, terpenes, alcohols, aldehydes, ketones, esters, and other metabolites. GC–TOF–MS revealed a total of 705 metabolites, including 91 benzene/phenylpropanoid compounds, 84 terpenoids, and 66 ester compounds. LC–MS (POS) revealed a total of 1093 metabolites, including benzene, 89 types of phenylpropanoid compounds, 67 types of terpenoid compounds, and 91 types of ester compounds; 302 metabolites were detected via LC–MS (NEG), including 25 types of benzene/phenylpropanoid compounds, 14 types of terpenoid compounds, and 19 kinds of ester compounds. Among them, benzene/phenylpropanoid compounds were the most abundant, accounting for 12.91, 8.14, and 8.28% of the total metabolites detected via GC–TOF–MS, LC–MS (POS), and LC–MS (NEG), respectively. Principal component analysis (PCA) of the quantification of the metabolites from the petals of the two different rhododendron varieties at four developmental stages revealed that all the biological replicates were grouped together, which indicated a good correlation between replicates and that our data were highly reliable. Moreover, the results showed that the samples of different varieties (NW and YJ) and at different stages (1, 2, 3, and 4) were scattered, indicating that there were differences between the different samples (Fig. 1c, d, e). Separation between the developmental stages could be explained by first principal component (PC1), while separation according to rhododendron type could be explained by the second principal component (PC2).

**Table 1:** Metabolites detected in rhododendron petals

	Benzenes/Phenylpropanoids	Terpenes	Alcohols	Aldehydes	Ketones	Esters	Others
GC–MS	91	84	101	33	68	66	262
LC–MS (POS)	89	67	49	24	141	91	632
LC–MS (NEG)	25	14	5	1	37	19	201

**Fig. 2:**
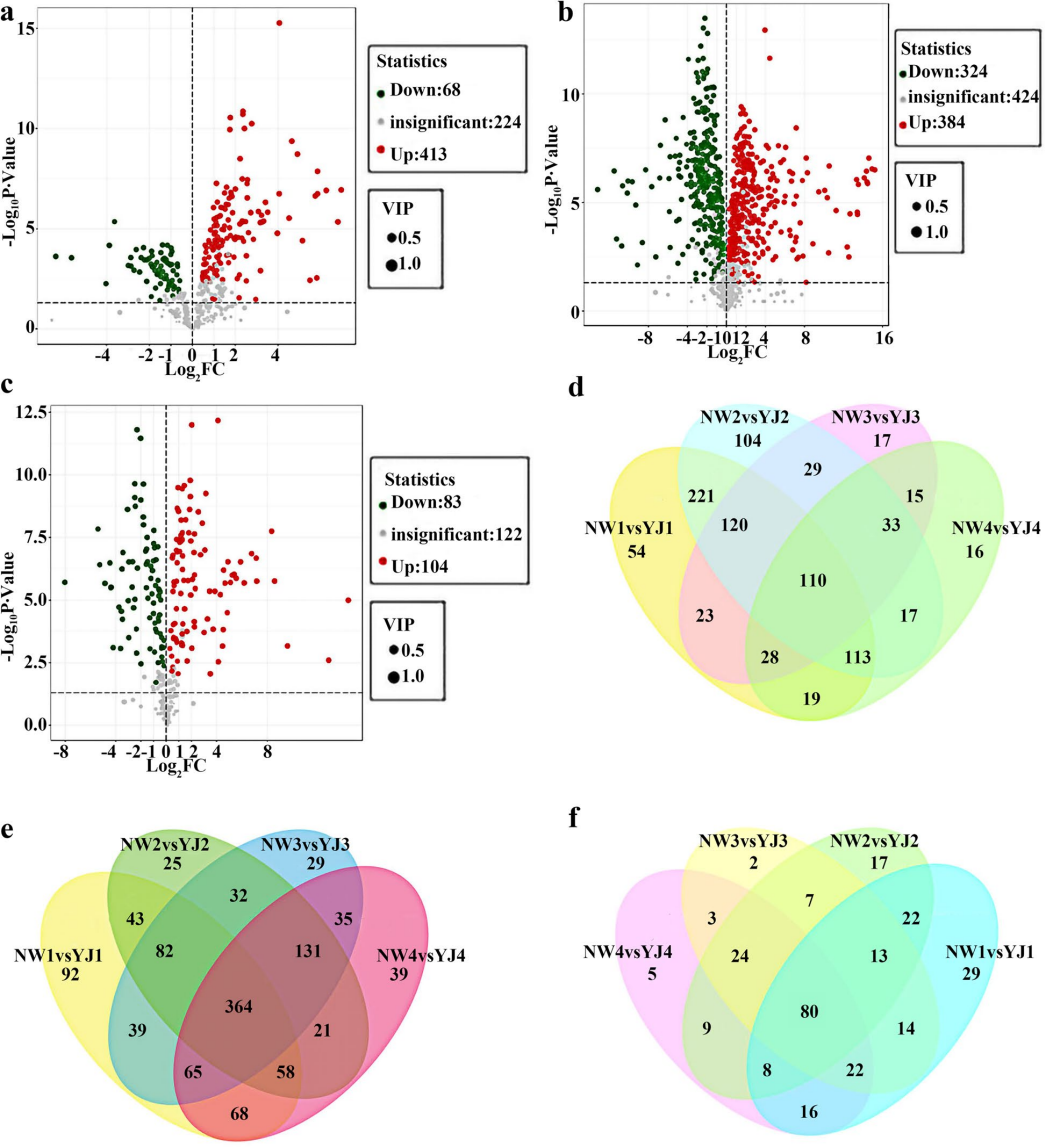
Identification of differentially accumulated metabolites in petals of different rhododendron varieties (NW and YJ) at the same developmental stage. **a** Volcano plot of differentially accumulated metabolites in the NW1 vs. YJ1 comparison group, as detected by GC–TOF–MS. **b** Volcano plot of differentially accumulated metabolites in the NW1 vs. YJ1 comparison group, as detected by LC–MS (POS). **c** Volcano plot of differentially accumulated metabolites in the NW1 vs. YJ1 comparison group, as detected by LC–MS (NEG). **d** Venn diagram of common differentially accumulated metabolites detected by GC–TOF–MS. **e** Venn diagram of common differentially accumulated metabolites detected by LC–MS (POS). **f** Venn diagram of common differentially accumulated metabolites detected by LC–MS (NEG)

Metabolites that differentially accumulated between comparison groups were identified based on the P value and the variable importance in the projection (VIP). There were significant differences in metabolites between the different varieties at the same developmental stage. For the NW1 vs. YJ1 comparison group, 481 differentially accumulated metabolites were identified by GC–TOF–MS, of which 413 and 68 of these metabolites increased and decreased in abundance, respectively (Fig. 2a). Similarly, 708 differentially accumulated metabolites were screened by LC–MS (POS), of which 384 and 324 increased and decreased in abundance, respectively (Fig. 2b). A total of 187 differentially accumulated metabolites were screened by LC–MS (NEG), of which 104 and 83 increased and decreased in abundance, respectively (Fig. 2c). Similarly, in the NW2 vs. YJ2 comparison group, NW3 vs. YJ3, and NW4 vs. YJ4 comparison groups, 526, 145, and 227 differentially accumulated metabolites were detected by GC–TOF–MS, respectively, of which 474, 72, and 81 increased in abundance and 52, 73, and 146 decreased in abundance (Additional file 1: Figs. S1, 2, 3). In total, 651, 660, and 672 differentially accumulated metabolites were detected by LC–MS (POS), of which 363, 336, and 267 increased in abundance and 288, 324, and decreased in abundance, respectively (Additional file 1: Figs. S4, 5, 6). In total, 159, 145, and 150 differentially accumulated metabolites were detected by LC–MS (NEG), respectively, of which 79, 72, and 78 increased in abundance and 80, 73, and 72 decreased in abundance (Additional file 1: Figs. S7, 8, and 9). GC–TOF–MS revealed that there were 110 differentially accumulated metabolites in common between *R. fortunei* and *Rhododendron* ‘Nova Zembla’ at the same developmental stage (NW1 vs. YJ1, NW2 vs. YJ2, NW3 vs. YJ3, and NW4 vs. YJ4) (Fig. 2d); 364 differentially accumulated metabolites were detected by LC–MS (POS) (Fig. 2e), and 80 differentially accumulated metabolites were detected LC–MS (NEG) (Fig. 2f). Compared with comparison group comprising different rhododendron varieties at the same developmental stage, the NW2 vs. YJ2 comparison group had the most differentially accumulated volatile metabolites, and the NW1 vs. YJ1 group had the most differentially accumulated nonvolatile metabolites.

**Fig. 3:**
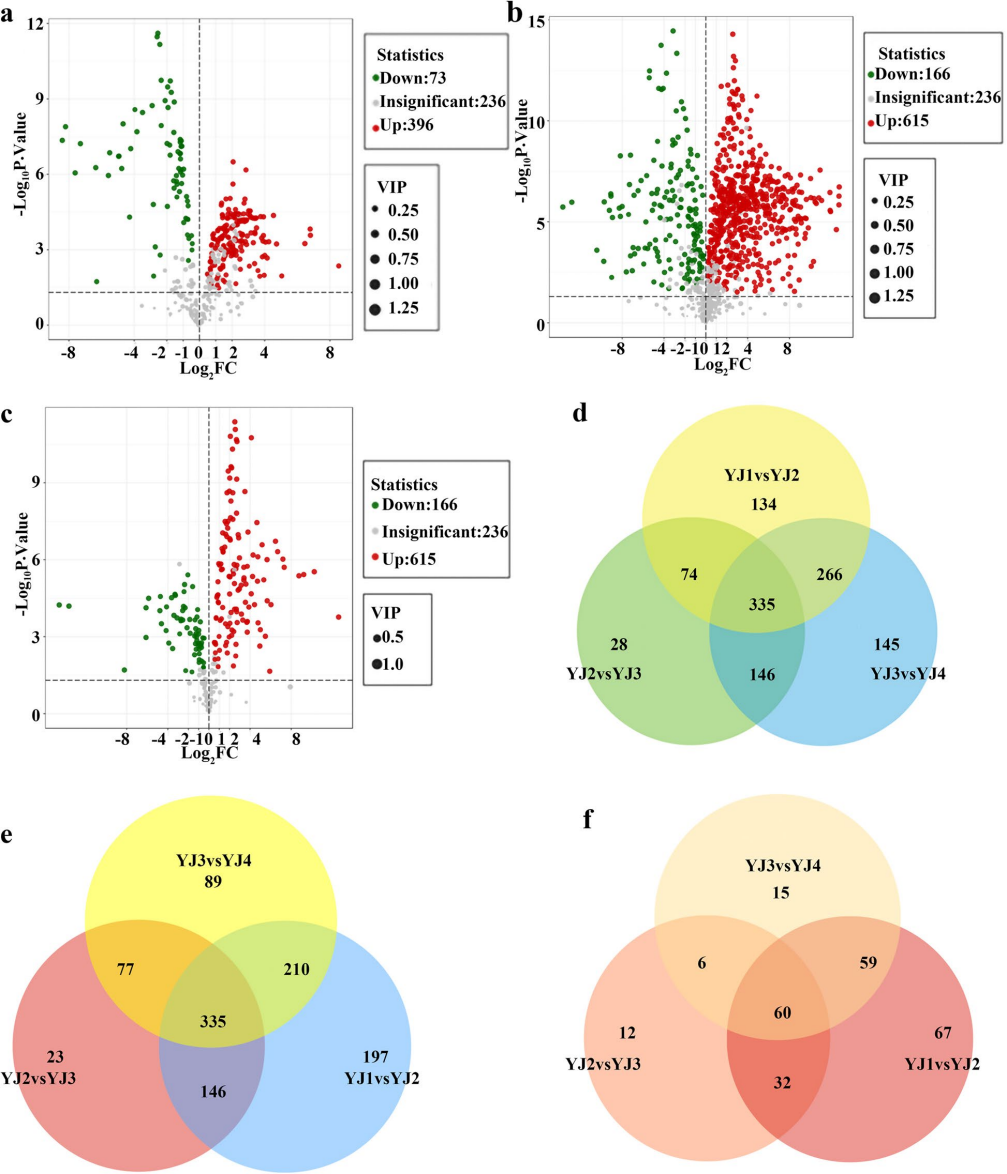
Identification of differentially accumulated metabolites in YJ at different developmental stages. **a** Volcano plot of differentially accumulated metabolites in the YJ1 vs. YJ2 comparison group, as detected by GC–TOF–MS. **b** Volcano plot of differentially accumulated metabolites in the YJ1 vs. YJ2 comparison group, as detected by LC–MS (POS). **c** Volcano plot of differentially accumulated metabolites in the YJ1 vs. YJ2 comparison group, as detected by LC–MS (NEG). **d** Venn diagram of common differentially accumulated metabolites detected by GC–TOF–MS. **e** Venn diagram of common differentially accumulated metabolites detected by LC–MS (POS). **f** Venn diagram of common differentially accumulated metabolites detected by LC–MS (NEG)

The metabolites of YJ also varied greatly at different developmental stages. For the YJ1 vs. YJ2 comparison group, a total of 469 differentially accumulated metabolites, including 396 and 73 that increased and decreased in abundance, respectively, were detected by GC–TOF–MS (Fig. 3a); 781 differentially accumulated metabolites were detected by LC–MS (POS), of which 615 and 166 increased and decreased in abundance, respectively (Fig. 3b); and 196 differentially accumulated metabolites were detected by LC–MS (NEG), of which 130 and 66 increased and decreased in abundance, respectively (Fig. 3c). We identified 224 differentially accumulated metabolites, including 107 and 117 metabolites that increased and decreased in abundance, respectively, in the YJ2 vs. YJ3 comparison group via GC–TOF–MS (Additional file 1: Fig. S10), and 448 differentially accumulated metabolites were screened by LC–MS (POS), of which 322 and 166 increased and decreased in abundance, respectively (Additional file 1: Fig. S11). In addition, 101 differentially accumulated metabolites were screened by LC–MS (NEG), of which 78 and 23 increased and decreased in abundance, respectively (Additional file 1: Fig. S12). For the YJ3 vs. YJ4 comparison group, a total of 448 differentially accumulated metabolites, including 77 and 371 that increased and decreased in abundance, respectively, were detected by GC–TOF–MS (Additional file 1: Fig. S13); 610 differentially accumulated metabolites were screened by LC–MS (POS), of which 179 and 431 increased and decreased in abundance, respectively (Additional file 1: Fig. S14); and 122 differentially accumulated metabolites were screened by LC–MS (NEG), of which 58 and 64 increased and decreased in abundance, respectively (Additional file 1: Fig. S15). GC–TOF–MS revealed 204 differentially accumulated metabolites in common among the three comparison groups (YJ1 vs. YJ2, YJ2 vs. YJ3 and YJ3 vs. YJ4) at adjacent developmental stages of YJ (Fig. 3d), 335 were detected by LC–MS (POS) (Fig. 3e), and 60 were detected by LC–MS (NEG) (Fig. 3f). Compared with the group comprising the same variety but different developmental stages, the YJ1 vs. YJ2 comparison group presented the greatest number of differentially accumulated metabolites.

### Analysis of differentially accumulated benzene/phenylpropanoid metabolites

**Fig. 4:**
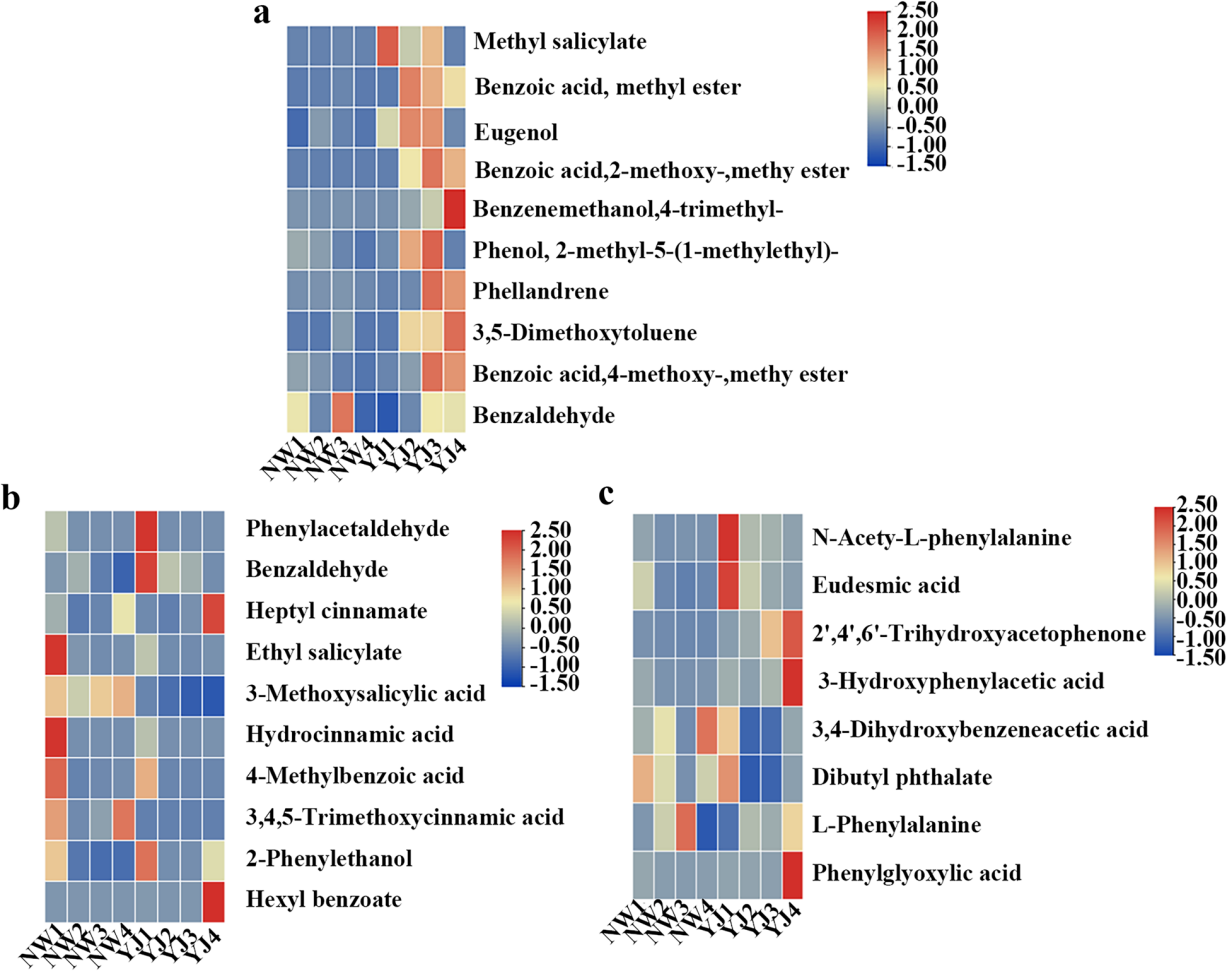
Changes in benzene/phenylpropanoid contents in NW and YJ. **a** Benzenes/phenylpropanoids detected by GC–TOF–MS, **b** Benzenes/phenylpropanoids detected by LC–MS (POS), **c** Benzenes/phenylpropanoids detected by LC–MS (NEG)

According to the results of our multivariate statistical analysis of the metabolome in ‘Multivariate statistical analysis of the metabolome’, benzenes/phenylpropanoids were the most abundant metabolites detected, and further analysis of benzene/phenylpropanoid metabolites was carried out. Differentially accumulated metabolites detected via GC–MS, LC–MS (POS), and LC–MS (NEG) in different developmental stages and between varieties were screened, and a heatmap was constructed according to the relative quantitative values of the samples to visualize the data (Fig. 4a, b, c). Nine of the 10 benzene/phenylpropanoid volatiles accumulated to a large degree in YJ but hardly accumulated in NW, and only benzaldehyde accumulated in both. Of these, only methyl salicylate accumulated the most in YJ1; two metabolites, benzoic acid methyl ester and eugenol, accumulated the most in YJ2; four metabolites, benzoic acid 2-methoxy-methyl ester, phenols, 2-methyl-5-(1-methylethyl)-, phellandrene, and benzoic acid 4-methoxy-methyl ester, accumulated the most in YJ3; and benzene ethanol and 4-trimethyl- and 3,5-dimethoxytoluene accumulated the most in YJ4. Six of the nine benzene/phenylpropanoid volatiles accumulated in YJ and showed an accumulation trend of first increasing and then decreasing; four types peaked in YJ3, and two types peaked in YJ2. Among the 10 various nonvolatile benzene/phenylpropanoid metabolites detected by LC–MS (POS), five metabolites, including ethyl salicylate, 3-methoxysalicylic acid, hydrocinnamic acid, 4-methylbenzoic acid, and 3,4,5-trimethoxycinnamic acid, accumulated more in NW than in YJ; however, excluding 3-methoxysalicylic acid and 3,4,5-trimethoxycinnamic acid, the other three metabolites tended to decrease with time. Among the five metabolites that accumulated the most in YJ, phenylacetaldehyde, benzaldehyde and 2-phenylethanol accumulated the most in YJ1, and heptyl cinnamate and hexyl benzoate accumulated the most in YJ4. There was almost no accumulation of these metabolites in YJ2 and YJ3. Of the eight various nonvolatile benzene/phenylpropanoid metabolites detected by LC–MS (NEG), six metabolites, including N-acetyl-L-phenylalanine, eudesmic acid, 2′,4′,6′-trihydroxyacetophenone, 3-hydroxyphenylacetic acid, dibutyl phthalate and phenylglyoxylic acid, accumulated more in YJ than in NW. These metabolites accumulated the most in YJ1 or YJ4; only 2′,4′,6′-trihydroxyacetophenone accumulated in YJ3, and the others did not accumulate in YJ2 or YJ3.

### Transcriptomic analysis

#### Overview of the transcriptomic response

Using the DNBSEQ platform for sequencing, we obtained a total of 153.37 Gb of sequencing data after assembly and deredundancy were performed. A total of 140,527 unigenes were identified, and the total length, average length, N50 and GC content were 168,644,638 bp, 1200 bp, 1887 bp, and 44.65%, respectively. The number of unigenes with a length of 300–400 bp constituted the greatest proportion – 12,714 unigenes, accounting for 15.01%. With an increase in length, the number of unigenes decreased, and the number of unigenes longer than 3000 bp increased (Additional file 1: Fig. S16). The clean read Q20 values of each group of samples was greater than 96%, and the clean read Q30 value of each group was greater than 90% (Additional file 2: Table S1). Therefore, the quality of the sequencing data was high and met the requirements of subsequent analysis (Additional file 2: Table S2). The results of our PCA showed that all biological replicates clustered together, indicating a good correlation between replicates. Moreover, we observed that the samples of different varieties (NW and YJ) and at different developmental stages (A, B, C, and D) were scattered, indicating that there were differences between different samples (Additional file 1: Fig. S17). Separation between the developmental stages could be explained by PC1, while separation according to the rhododendron type could be explained by PC2. The unigene sequences were compared to sequence data within seven functional databases for annotations, and 102,107 (nonredundant (NR) protein: 72.66%), 77,226 (nucleotide (NT): 54.95%), 77,392 (SwissProt: 55.07%), 80,356 (EuKaryotic Orthologous Groups (KOG): 57.18%), 79,858 (Kyoto Encyclopedia of Genes and Genomes (KEGG): 56.83%), 74,026 (Gene Ontology (GO): 52.68%), and 76,946 (Pfam: 54.76%) unigenes were functionally annotated.

#### Identification of DEGs

**Fig. 5:**
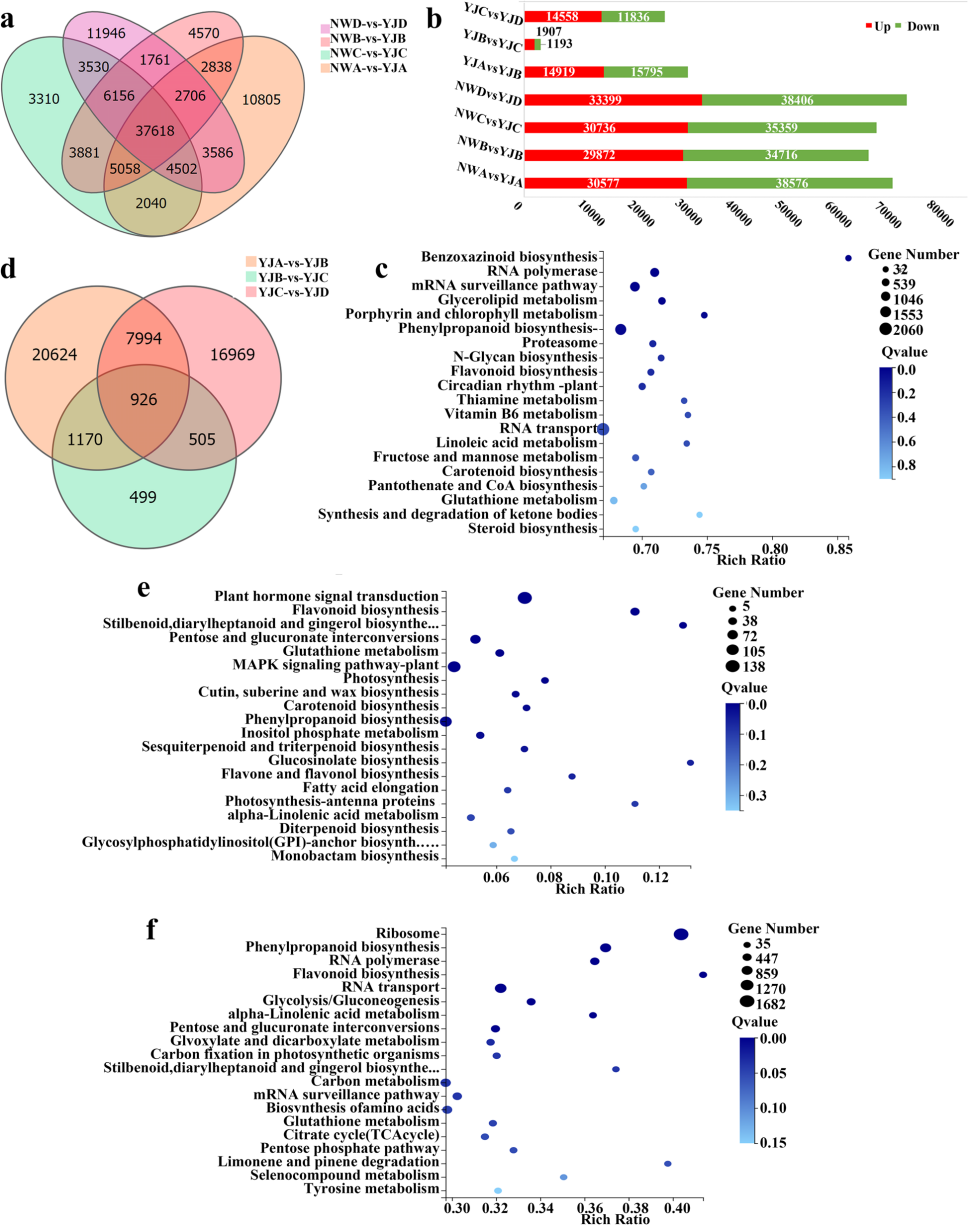
Identification of DEGs by RNA-seq analysis. **A** Venn diagram of DEGs shared between different varieties of *Rhododendron* (NW and YJ) at different developmental stages, **b** Number of DEGs, **c** Top 20 KEGG pathways with the most significant DEG enrichment in the NWD vs. YJD comparison group, **d** Venn diagram of common DEGs within YJ at different developmental stages (ABCD), **e** Top 20 KEGG pathways enriched in DEGs in the YJB vs. YJC comparison group, **f** Top 20 KEGG pathways enriched in DEGs in the YJC vs. YJD comparison group

To identify the key genes differentially expressed in the different varieties of *Rhododendron*, the DEGs in each of the NWA vs. YJA, NWB vs. YJB, NWC vs. YJC, and NWD vs. YJD comparison groups were analysed, and it was found that there were 37,618 DEGs in common among these four groups (Fig. 5a). For the NWA vs. YJA comparison group, a total of 69,153 DEGs were detected, namely, 30,577 upregulated genes and 38,576 downregulated genes. Similarly, the DEGs in the NWB vs. YJB, NWC vs. YJC, and NWD vs. YJD comparison group were analysed. There were 64,588, 66,095, and 71,805 DEGs, respectively, of which 29,872, 30,736, and 33,399 genes were upregulated and of which 34,716, 35,359, and 38,406 genes were downregulated (Fig. 5b). Among them, the NWD vs. YJD comparison group exhibited the largest difference in gene expression, whereas the NWB vs. YJB comparison group had relatively few DEGs. To identify the metabolic pathways active during petal development, the genes that were differentially expressed between groups were subjected to KEGG pathway enrichment analysis, and the top 20 KEGG pathways significantly enriched are shown in Fig. 5c and Additional file 1: Figs. S18, 19, and 20. The top three significantly enriched pathways in the NWA vs. YJA and NWB vs. YJB comparison groups were associated with plant–pathogen interactions, plant hormone signal transduction and endocytosis (Additional file 1: Figs. S18, 19). The top three significantly enriched pathways in the NWC vs. YJC comparison group were associated with plant hormone signal transduction, plant–pathogen interactions, and ubiquitin-mediated proteolysis (Additional file 1: Fig. S20). The top three significantly enriched pathways in the NWD vs. YJD comparison group were RNA transport, phenylpropanoid biosynthesis, and the Mrna surveillance pathway. Among them, 1640 DEGs in the NWD vs. YJD comparison group were enriched in the phenylpropanoid biosynthesis pathway, which is related to the synthesis of floral aromatic compounds (Fig. 5c).

To identify the key genes differentially expressed between the different developmental stages of YJ, the DEGs among the YJA vs. YJB, YJB vs. YJC, and YJC vs. YJD comparison groups were analysed, and it was found that there were 926 DEGs in common among the three groups (Fig. 5d). For the YJA vs. YJB comparison group, a total of 30,714 DEGs were detected, namely, 14,919 upregulated genes and 15,795 downregulated genes (Fig. 5b). Similarly, the DEGs in the YJB vs. YJC and YJC vs. YJD comparison groups were analysed, and there were 3100 and 26,394 DEGs, respectively, of which 1907 and 14,558 genes, respectively, were upregulated and 1193 and 11,836 genes, respectively, were downregulated (Fig. 5b). Among them, the YJB vs. JC comparison group had fewer DEGs than did the YJA vs. YJB and YJC vs. YJD comparison group, and the YJA vs. YJB comparison group had the most DEGs. Further division of the DEGs between groups was performed via KEGG pathway enrichment analysis, and the top 20 significantly enriched KEGG pathways are shown in Fig. 5e and f and Additional file 1: Fig. S21. The top three significantly enriched pathways in the YJA vs. YJB comparison group were plant–pathogen interaction, plant hormone signal transduction and MAPK signalling pathway-plant (Additional file 1: Fig. S21); the top three significantly enriched pathways in the YJB vs. YJC comparison group were plant hormone signal transduction, MAPK signalling pathway-plant and phenylpropanoid biosynthesis; and the top three significantly enriched pathways in the YJC vs. YJD comparison group were ribosome, phenylpropanoid biosynthesis, and RNA transport (Fig. 5f). Among these comparison groups, the YJB vs. YJC and YJC vs. YJD comparison groups had 97 and 867 DEGs, respectively, enriched in the phenylpropanoid biosynthesis pathway (Fig. 5e, f). Because the phenylpropanoid biosynthesis pathway is involved in regulating floral aromatic compounds [10], the DEGs enriched in the phenylpropanoid biosynthesis pathway were further analysed.

### Screening of DEGs involved in the phenylpropanoid pathway

According to the identification and KEGG enrichment analysis results of the DEGs, those in the NWD vs. YJD, YJB vs. YJC, and YJC vs. YJD comparison group were found to be enriched in the phenylpropanoid biosynthesis pathway. After consulting the literature on the study of floral genes involved in the phenylpropanoid pathway, we screened nine DEGs for analysis (Additional file 2: Table S3): phenylalanine ammonia lyase (*PAL*), cinnamate 4-hydroxylase (*C4H*), 4-coumaroyl-CoA ligase (*4CL*), salicylic acid carboxyl methyl transferase (*SAMT*), caffeoyl-CoA 3-O-methyltransferase (*CCoAOMT*), cinnamoyl-CoA reductase (CCR), cinnamyl alcohol dehydrogenase (*CAD*), eugenol synthase (*EGS*), and benzaldehyde dehydrogenase (*BALDH*). According to the transcriptome sequencing results, RSEM was used to calculate the gene expression levels in each sample, and a heatmap was constructed to visualize the expression levels of the abovementioned genes in the petals of the different rhododendron varieties at different developmental stages to visualize the results. Figure 7 shows that only four unigenes were highly expressed in NW, and the expression levels of these nine genes in YJ were always higher than those in NW. These results are consistent with the results of our metabolomic analysis, indicating that phenylpropanoids were abundant in YJ but not in NW. Figure 7 shows the synthesis pathway of volatile phenylpropanoids; *4CL*, *CCR*, and *CAD* were expressed in in YJ at each developmental stage, and the expression of each unigene was downregulated in YJC. The expression of *PAL* was downregulated in YJB and YJC, that of *CCoAOMT* was downregulated in YJC, and that of *C4H* was downregulated in YJD. *EGS* expression was upregulated in YJB and high in YJB and YJC, *SAMT* expression was upregulated in YJD, and *BALDH* was highly expressed in YJC and YJD.

**Fig. 6:**
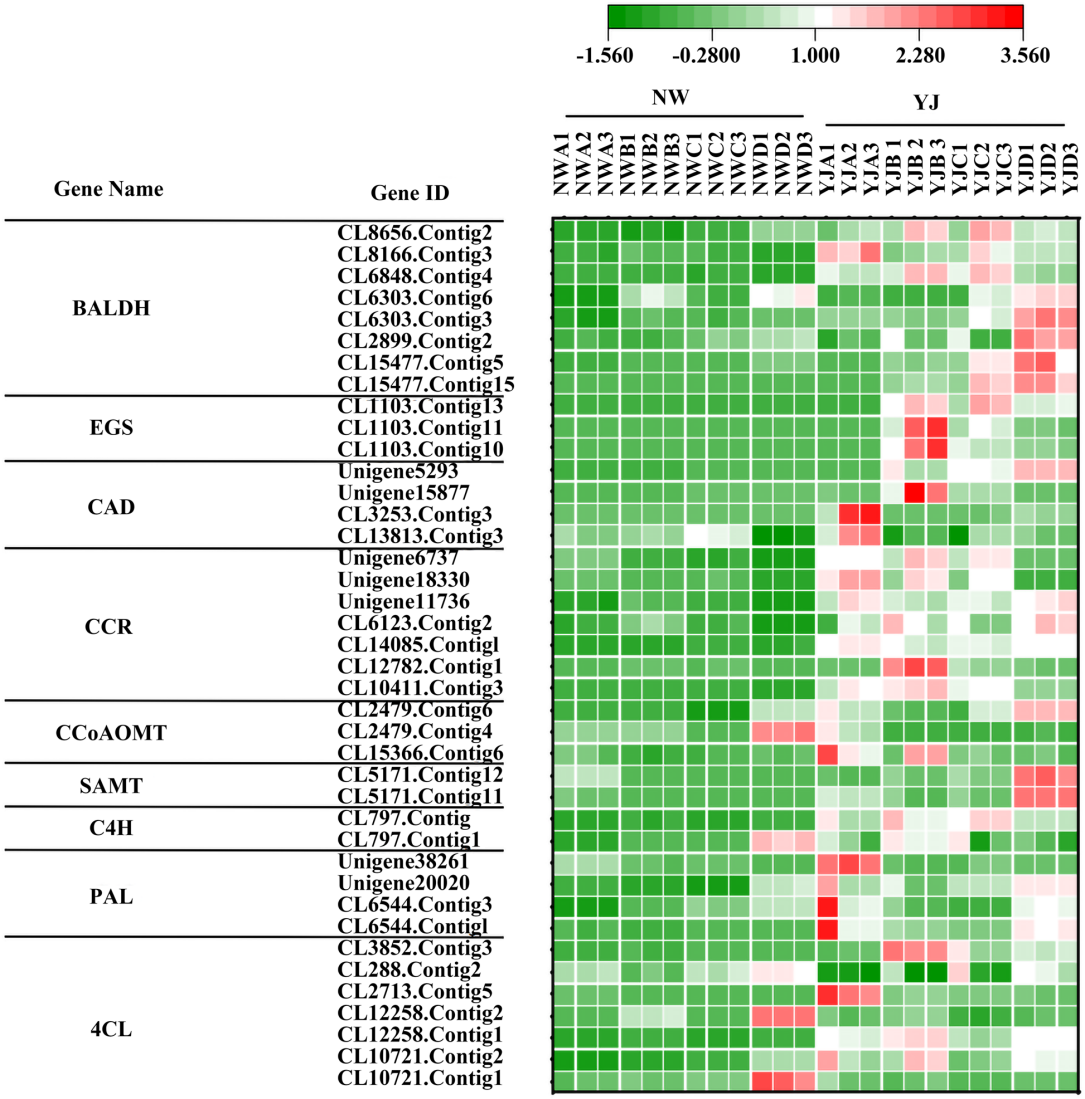
Relative changes in the expression of candidate genes in different rhododendron varieties (NW and YJ) at different developmental stages (**A**, **B**, **C**, **D**)

To validate the RNA sequencing (RNA-seq) data, we conducted qRT–PCR analyses of the nine candidate genes. The expression levels of the candidate genes in the petals of NW and YJ were measured at stages A, B, C, and D. The transcript profiles of all the selected genes were highly consistent with those detected from the RNA-seq data (Fig. 8). The expression levels of these nine genes in YJ were always higher than those in NW, demonstrating that the RNA-seq data and DEG analysis results in this study were reliable (Fig. 6).

### Correlation analysis of DEGs and differentially accumulated benzene/phenylpropanoid metabolites

To understand the relationships between genes and metabolites during flowering, correlation analysis between diethylene glycol and phenylpropanoid metabolites and 9 select genes involved in the phenylpropanoid pathway was carried out. NW1 vs. YJ1 analysis showed that benzaldehyde content was significantly positively correlated with *PAL* expression and significantly negatively correlated with *C4H*, *4CL*, and *BALDH* expression; eugenol content was significantly positively correlated with *PAL* expression and significantly negatively correlated with *CAD* expression; benzoic acid methyl ester content was significantly negatively correlated with *BALDH* expression; and methyl salicylate content was significantly negatively correlated with *4CL* and *CAD* expression and significantly positively correlated with *BALDH* and *CCR* expression (Fig. 9a). Hexyl benzoate, 2-phenylethanol, benzaldehyde, and phenylacetaldehyde contents were significantly negatively correlated with *CCR* expression and significantly positively correlated with *C4H*, *4CL*, and *BALDH* expression; 4-methylbenzoic acid, ethyl salicylate, and heptyl cinnamate contents were significantly positively correlated with *4CL* and *CCoAOMT* expression and significantly negatively correlated with *BALDH* and *CCR* expression (Fig. 9b). 2,4,6-Hydroxyacetophenone content was significantly positively correlated with *C4H*, *4CL*, and *CCoAOMT* expression and significantly negatively correlated with *CCR* expression; 3,4,5-trimethoxybenzoic acid and N-acetyl-L-phenylalanine contents were significantly negatively correlated with *4CL* expression and significantly positively correlated with *BALDH* and *CCR* expression (Fig. 9c). NW2 vs. YJ2 analysis showed that methyl salicylate, benzoic acid methyl ester, and eugenol contents were significantly negatively correlated with *PAL* and *BALDH* expression and significantly positively correlated with *4CL*, *CAD*, *EGS*, and *CCR* expression (Additional file 1: Fig. S22a). The contents of multiple metabolites were significantly positively correlated with *PAL* and *EGS* expression and significantly negatively correlated with *SAMT* expression (Additional file 1: Fig. S22b, c). NW3 vs. YJ3 analysis showed that the contents of various metabolites were significantly positively correlated with *C4H*, *EGS*, and *CCoAOMT* expression and significantly negatively correlated with *CCR* expression (Additional file 1: Fig. S23a). Phenylethanol and heptyl cinnamate contents were significantly positively correlated with *PAL*, *EGS*, *BALDH* and *CCR* expression and significantly negatively correlated with *4CL* expression, and 4-methylbenzoic acid and phenylacetaldehyde contents were significantly positively correlated with *SAMT*, *BALDH* and *CCR* expression (Additional file 1: Fig. S23b). The contents of a variety of metabolites were significantly positively correlated with *4CL* and *CCR* expression and significantly negatively correlated with *BALDH* expression (Additional file 1: Fig. S23c). NW4 vs. YJ4 analysis showed that the contents of various metabolites were significantly positively correlated with *EGS* expression and significantly negatively correlated with *4CL*, *BALDH*, and *CCR* expression (Additional file 1: Fig. S24a). The contents of multiple metabolites were significantly positively correlated with *PAL*, *C4H*, *EGS*, and *CAD* expression and significantly negatively correlated with *4CL* and *CCR* expression (Additional file 1: Fig. S24b). The contents of various metabolites were significantly positively correlated with *EGS* expression and significantly negatively correlated with *CCR* expression (Additional file 1: Fig. S24c). YJ1 vs. YJ2 analysis showed that the contents of many metabolites were significantly negatively correlated with *BALDH* expression and significantly positively correlated with *4CL*, *EGS*, and *CCR* expression (Additional file 1: Fig. S25a). The contents of multiple metabolites were significantly negatively correlated with *SAMT*, *4CL*, *EGS*, *CCR*, *PAL*, and *CAD* expression and significantly positively correlated with *BALDH* and *CCoAOMT* expression (Additional files 1: Figs. S25b, c). YJ2 vs. YJ3 analysis showed that benzoic acid methyl ester content was significantly negatively correlated with *PAL* expression; benzaldehyde content was significantly negatively correlated with *4CL* and *CCR* expression and significantly positively correlated with *BALDH* expression; and methyl salicylate content was significantly positively correlated with *BALDH* expression (Additional file 1: Fig. S26a). Heptyl cinnamate content was significantly positively correlated with *BALDH* expression; 3-hydroxyphenylacetic acid and 2,4,6-trihydroxyacetophenone contents were significantly positively correlated with *PAL* expression and significantly negatively correlated with *CCR* expression; and eudesmic acid and N-acetyl-L-phenylalanine contents were significantly negatively correlated with *BALDH* expression and significantly positively correlated with *CCR* expression (Additional file 1: Fig. S26b, c). YJ3 vs. YJ4 analysis showed that 3,5-dimethoxytoluene content was significantly negatively correlated with *4CL* and *CCR* expression and significantly positively correlated with *BALDH* expression; the contents of phenol, 4-methyl-5-(1-methylethyl), benzenemethanol, 4-trimethyl, and eugenol were significantly positively correlated with *BALDH*, *4CL*, *CAD*, and *EGS* expression and significantly negatively correlated with CCR expression (Additional file 1: Fig. S27a). Hexyl benzoate content was significantly positively correlated with PAL, 4CL, CAD and BALDH expression and significantly negatively correlated with CCR expression; hydrocinnamic acid content was significantly negatively correlated with CAD expression; 3-methoxysalicylic acid content was significantly positively correlated with BALDH and CCR expression; phenylglyoxylic acid and L-phenylalanine (Phe) contents were significantly negatively correlated with PAL, 4CL, CAD, EGS, BALDH, and CCR expression and were significantly positively correlated with *SAMT* expression; and 3,4 − dihydroxybenzeneacetic acid, 3-hydroxyphenylacetic acid, and 2,4,6-trihydroxyacetophenone contents were significantly positively correlated with PAL and CCR expression and significantly negatively correlated with EGS expression (Additional file 1: Fig. S27b, c). By analysing multiple groups of samples, we ultimately found that the expression of nine genes, namely, *SAMT*, *BALDH*, *CCoAOMT*, *CCR*, *EGS*, *4CL*, *C4H*, *CAD,* and *PAL*, was significantly correlated with the contents of volatile benzene aromatic compounds and was associated with the involvement of volatile benzene. The contents of precursor nonvolatile metabolites formed by similar metabolites were also significantly correlated, indicating that these 9 genes were involved in the regulation of YJ aroma. However, how these key genes are involved in the regulation of the YJ aroma still needs to be verified by experiments. In this study, the function of the *SAMT* gene was verified by overexpression.

### *RfSAMT* gene functional verification

#### Gene expression changes after transient overexpression of *RfSAMT*

The results of PCR amplification of the *SAMT* gene in *R. fortuneie* is shown in Fig. 10a. The WT flower buds and flower buds injected with an Agrobacterium infection solution containing an empty Pcambia1302 vector or an Agrobacterium infection solution containing a Pcambia1302-*SAMT* recombinant vector were removed after they bloomed; then, the stamens and pistils were removed, and the petals were used as experimental materials to measure the expression level of *RfSAMT*. The results showed that the expression of *RfSAMT* increased after transient overexpression of *RfSAMT*; the expression was 12.54 times higher in the overexpression group than in the WT control group (Fig. 10b).

#### Changes in the relative contents of the main floral aromatic compounds and methyl salicylate after transient overexpression of *RfSAMT*

Using the same experimental materials as described in ‘Gene expression changes after transient overexpression of *RfSAMT’*, we measured the changes in the relative contents of the main floral aromatic compounds and methyl salicylate in the different experimental groups. The results showed that after transient overexpression of *RfSAMT*, the main floral components in YJ were essentially unchanged, and the accumulation of only a few metabolites changed. The main volatile metabolites were (+)-bicyclosesquiphellandrene, methyl benzoate, salicylic acid (SA), methyl ester, α-terpineol, linalool, 3-carene, 3,5-dimethoxytoluene, sapinene, and β-bourbonene. (Additional file 2: Table S4). The relative levels of various metabolites increased, including those of linalool, methyl benzoate, α-terpineol, and 3-carene. The methyl salicylate content was 0.17 times that of the WT after transient overexpression, and the benzoic acid methyl ester content was 1.24 times that in the WT after transient overexpression (Fig. 10c).

## Discussion

Floral aroma is an important trait of plants. Floral aroma plays a key role in plant growth, development, and evolution, and it is one of the main quality traits affecting the ornamental and commercial value of *Rhododendron*. In this study, the transcriptome and metabolome were comprehensively analysed to increase our understanding of the molecular mechanism underlying the synthesis of benzene/phenylpropanoid-like compounds responsible for the fragrance of in YJ. Our findings contribute to future research on the molecular mechanism of aromatic compound synthesis in different rhododendron varieties and provide scientific reference data for aromatic breeding plant.

After terpenes, benzene/phenylpropanoid compounds constitute the second largest group of plant floral components. Phenylpropionic acid compounds take Phe) as a starting substrate and undergo a series of complex branch pathway reactions. The benzene/phenylpropanoid synthesis pathway is a branch of the phenylpropionic acid synthesis pathway, also known as the cinnamic acid pathway [25, 26]. In this study, the differentially accumulated metabolites in the petals of two rhododendron varieties and the dynamic changes in metabolites in the petals of YJ at four different developmental stages were analysed by means of nontargeted metabolomics. The detected metabolites were divided into seven categories, namely, benzene/phenylpropanoid compounds, terpenes, alcohols, aldehydes, ketones, esters, and other metabolites (Table 1). Among them, 705 types of VOCs were detected by GC–TOF–MS. Benzene/phenylpropanoid metabolites were the most frequently recorded VOCs and accounted for 12.9% (91 types) of the total VOCs (Table 1). The metabolome was further analysed, and 10 benzene/phenylpropanoids detected via GC–TOF–MS, LC–MS (POS) and LC–MS (NEG) were identified. Among the 10 benzene/phenylpropanoid volatiles, nine kinds of volatiles accumulated to a large degree in YJ but barely accumulated in NW, and six of these nine metabolites tended to increase first and then decrease in YJ. The accumulation trend, of which four varieties peaked in YJ3 and two varieties peaked in YJ2 (Fig. 4a), was consistent with the fragrance emitted by YJ while flowering, while NW flowers are unscented. In the YJ1 vs. YJ2 comparison group, there were 469, 781, and 196 differentially accumulated metabolites identified via GC–TOF–MS, LC–MS (POS), and LC–MS (NEG), respectively (Fig. 3a, b, c); these two developmental stages presented the most differentially accumulated metabolites. The YJ2 vs. YJ3 comparison group had the fewest differentially accumulated metabolites in the petals (Additional file 1: Fig. S10, S11, S12). The difference between the flower-bud stage and first-bloom stage was the largest; this stage corresponded to the greatest changes in the growth and development of YJ. Compared with group comprising the same developmental stage and the other variety, the NW2 vs. YJ2 comparison group had more differentially accumulated VOCs (Additional file 1: Fig. S1). It is speculated that floral VOCs begin to be released at this stage. The nonvolatile benzene/phenylpropanoid metabolites detected by LC–MS accumulated the most in YJ1 or YJ4 and hardly accumulated in YJ2 and YJ3 (Fig. 4b, c). These metabolites may be involved in the synthesis of floral VOCs and are the precursors of volatile benzene/phenylpropanoids. Biosynthesis of secondary metabolites relies on primary metabolic pathways to provide precursors, energy, and cofactors, thus requiring coordinated regulation of primary and secondary metabolic networks. However, to date, elucidation of how this coordination is achieved has remained untested [27]. To clarify whether there is a link between volatile and nonvolatile benzene/phenylpropanoids, further analysis at the genome-wide level using chromatin immunoprecipitation sequencing (ChIP-seq) is needed. Patrick et al. [27] used petunia as experimental material and used ChIP-seq to study the chromatin-level mechanisms regulating the formation of volatile compounds of primary metabolite precursors and secondary metabolites at the genome-wide level. The results showed that the metabolic network during flowering was regulated at the chromatin level through histone modifications such as H3K9ac, which promoted the activation of shikimate and phenylalanine synthesis pathways, provided precursors of primary metabolites, and promoted different secondary metabolic pathways. This activity ultimately led to the generation of floral VOCs.

**Fig. 7:**
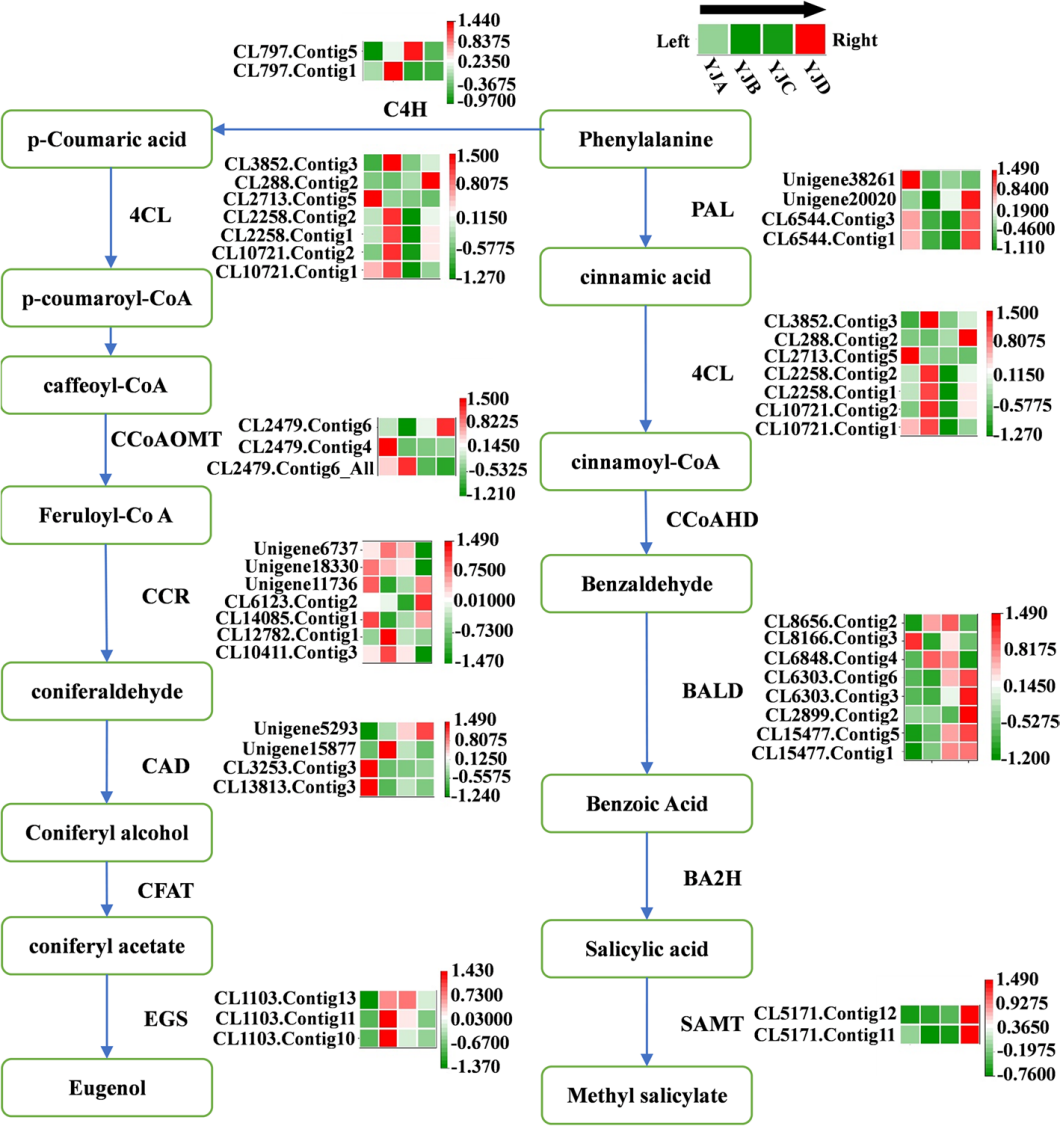
Differential expression of genes involved in the phenylpropanoid biosynthesis pathway in YJ at different developmental stages (**A**, **B**, **C**, **D**)

**Fig. 8:**
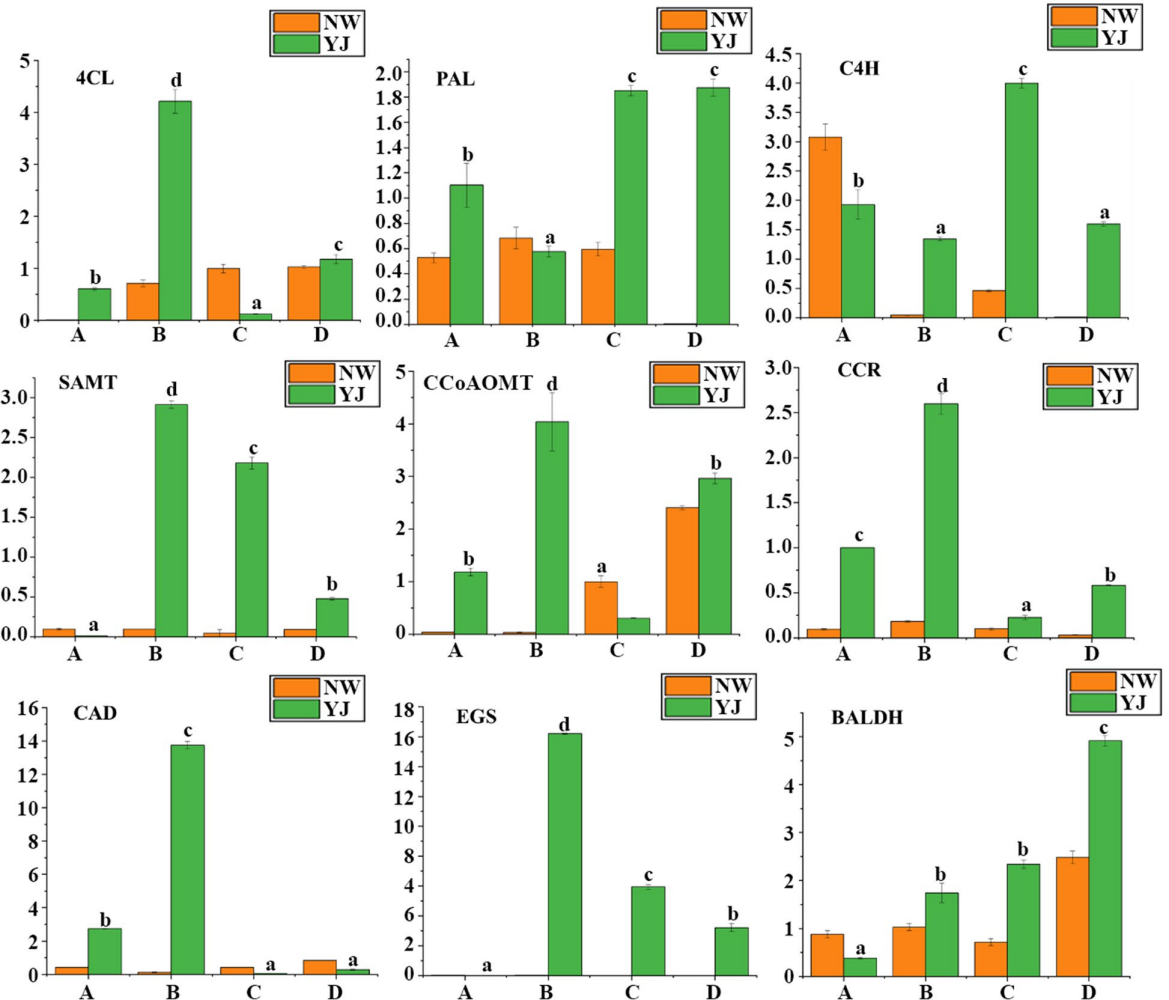
Histograms of qRT–PCR validation results of candidate genes. The y-axis shows relative gene expression levels, as determined by qRT –PCR. qRT –PCR gene relative expression data corresponding to NW (orange) and YJ (green). The data presented here are the averages of three replicates. The error bars represent the standard errors (Ses) (*n* = 3)

**Fig. 9:**
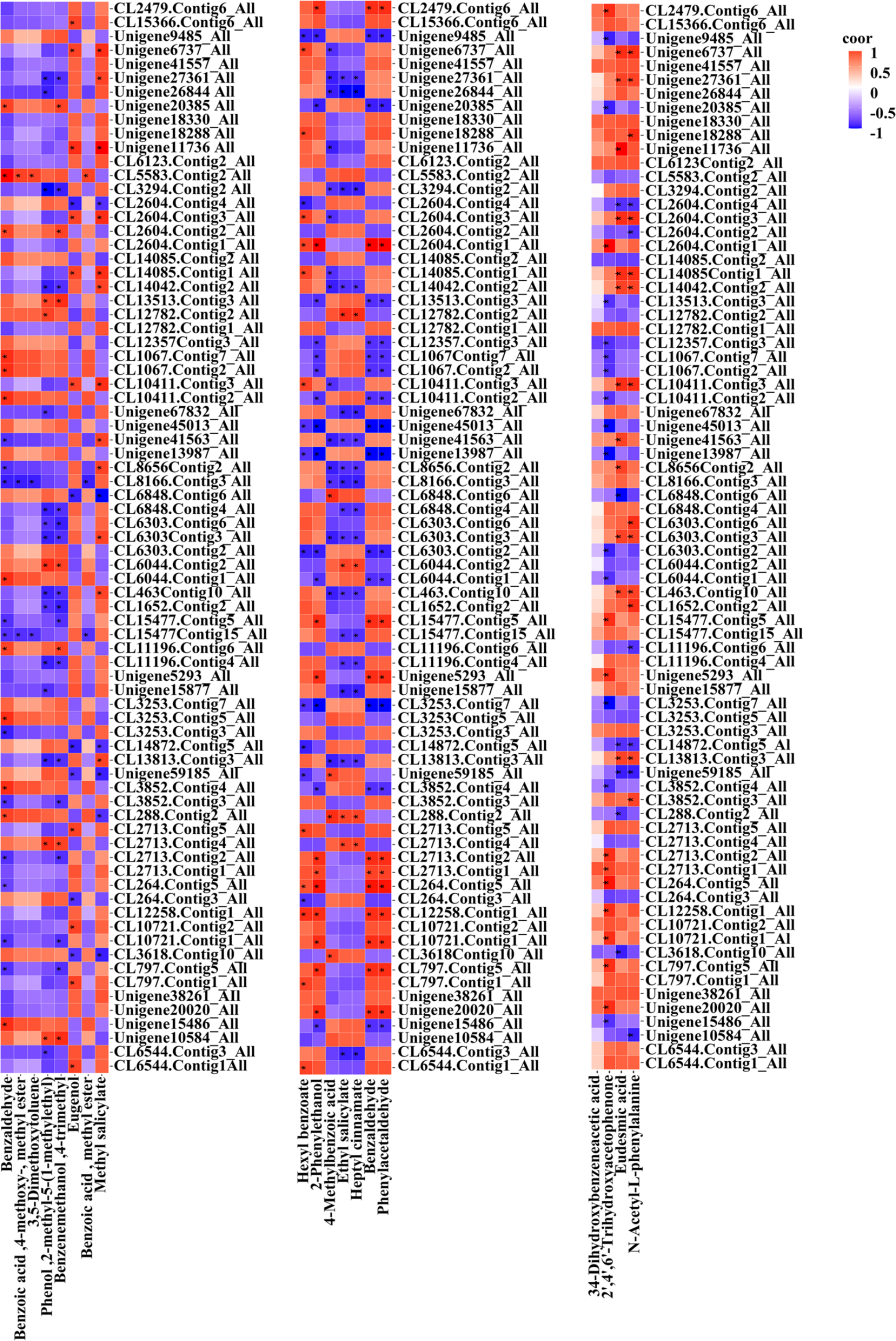
Correlation analysis of phenylpropanoid pathway candidate genes and benzene/phenylpropanoid metabolites in the NW1 vs. YJ1 comparison group. **A** GC–TOF–MS, **b** LC–MS (POS), **c** LC–MS (NEG)

In this study, transcriptomic data were analysed, and the results showed that there were differences in gene expression in YJ across different developmental stages. The difference in gene expression between the flower-bud stage and first-bloom stage was the largest, there were 30,714 DEGs in the YJA vs. YJB comparison group (Fig. 5b), and the difference in gene expression between the flower-bud stage and the full-bloom stage was the smallest. The YJB vs. YJC comparison group had 30,714 DEGs (Fig. 5b); the gene expression differences between the bloom stage and decay stages increased, and the YJC vs. YJD comparison group had 26,394 DEGs (Fig. 5b). The results of KEGG enrichment analysis showed that a large number of DEGs in the YJB vs. YJC (Fig. 5e) and YJC vs. YJD (Fig. 5f) comparison groups were enriched in the phenylpropanoid biosynthesis pathway. Therefore, it can be speculated that the expression of genes that regulate the synthesis of floral VOC precursor compounds is upregulated in the flower-bud stage and that the expression is downregulated from the bloom stage to the decay stage until the expression stops at the decay stage. The key genes regulating the synthesis of floral VOCs were upregulated at the first-bloom stage, and these genes were involved in the synthesis of floral VOCs in the bud stage, bloom stage and decay stage. The DEGs were further analysed, and nine candidate genes involved in the phenylpropanoid biosynthesis pathway were identified: *PAL*, *C4H*, *4CL*, *SAMT*, *CCoAOMT*, *CCR*, *CAD*, *EGS*, and *BALDH* (Additional file 2: Table S3). The transcriptomic data and qRT–PCR results showed that the expression levels of these nine genes increased in YJ and decreased in NW (Fig. 6 and Fig. 8), which suggests that these genes may be involved in the synthesis of floral VOCs responsible for fragrance. This is consistent with the result that benzene/phenylpropanoid volatiles accumulate in large amounts in YJ but hardly accumulate in NW (Fig. 4a). Correlation analysis will be performed in the future for a specific in-depth analysis of genes and metabolites to elucidate the biological processes underlying petal growth and floral VOC synthesis. In the present study, expression of the nine candidate genes was significantly correlated with benzene/phenylpropanoid metabolite contents, and these genes were found to be involved in the biosynthesis of VOCs in YJ. Among these genes, *4CL*, *CCR*, and *CAD* were expressed in YJ at each developmental stage, and all of them were downregulated in YJC (Fig. 7), which significantly negatively correlated with the contents of various benzene metabolites (Fig. 9, Additional file 1: Fig. S22–27). *4CL* plays an important role in the synthesis of benzene/phenylpropanoid compounds, which has also confirmed in agave, holy basil, and mulberry [28–31]. Zhang et al. [31] comprehensively analysed the endogenous volatile compounds in, transcriptome of, and enzymatic activities of plum blossoms, the results of which showed that *PmCAD1* plays an important role in the biosynthesis of cinnamyl alcohol in vitro. In the present study, the expression of *PAL* was downregulated in YJB and YJC (Fig. 7) was significantly negatively correlated with the contents of various benzene-like VOCs (Fig. 9a, Additional file 1: Fig. S22a-27a), and was significantly positively correlated with the contents of nonvolatile benzene-like metabolites (Fig. 9b, c, Additional file 1: Fig. S22b, c-27b, c). Verdonk et al. [32] performed a DNA microarray analysis and found that *PAL* regulates the production of petunia aromatic VOCs. In the present study, the expression of *CCoAOMT* was downregulated in YJC, the expression of *C4H* was downregulated in YJD, and *BALDH* was highly expressed in YJC and YJD (Fig. 7). The expression of all of these genes was significantly negatively correlated with the contents of a variety of volatile benzene/phenylpropanoid compounds and significantly positively correlated with the contents of a variety of nonvolatile compounds (Fig. 9, Additional file 1: Fig. S22–27). *EGS* was upregulated in YJB, highly expressed in YJB and YJC (Fig. 7) and was significantly positively correlated with benzene VOCs (Fig. 9a, Additional file 1: Fig. S22a-27a). *EGS* regulates the synthesis of benzene/phenylpropanoid compounds and plays a crucial role in the synthesis of floral VOCs; these findings have been confirmed in *Rosa rugosa*, *Albizia odoratissima*, *Gymnadenia conopsea*, *Dudleya densiflora*, and *Petunia hybrida* [33–35]. The expression of *SAMT* was upregulated in YJD (Fig. 7) and was significantly negatively correlated with benzene VOCs content (Fig. 9a, Additional file 1: Fig. S22a-27a). It has been confirmed that *SAMT* is a key gene regulating the synthesis of floral aromatic VOCs in various plant species, such as *Clarkia amoena*, *Lilium*, *Jasminum sambac*, and *Eriobotrya japonica* [36–41]. The release of methyl salicylate peaked at the flower-bud stage and remained stable throughout the floral growth and developmental stages, and *SAMT* expression was downregulated after the petals opened.

**Fig. 10:**
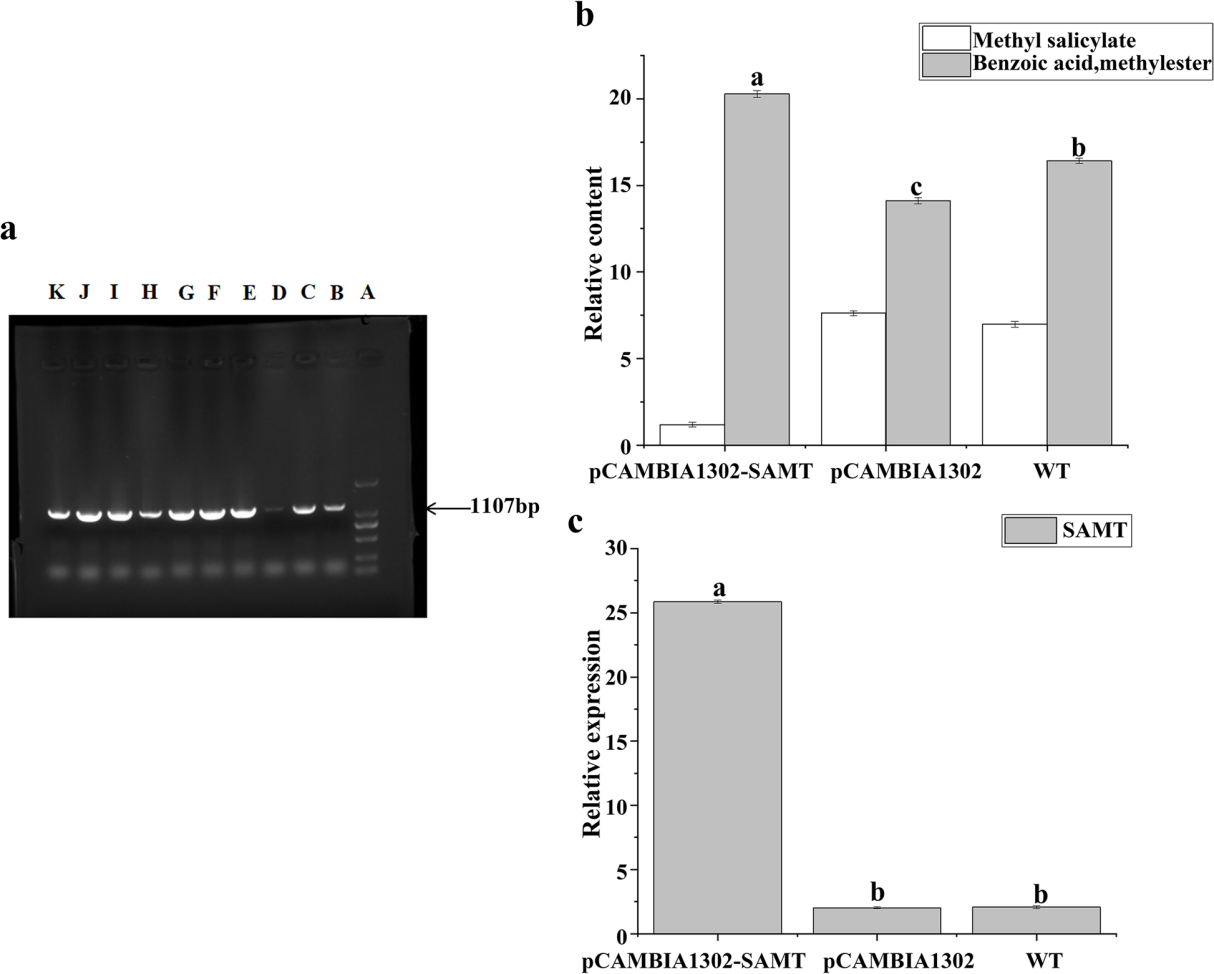
*RfSAMT* gene functional verification. The different lowercase letters indicate significant differences (*P* ≤ 0.05). Pcambia1302: empty overexpression plasmid control group; Pcambia1302-*SAMT*: recombinant plasmid treatment group in which the *RfSAMT* target gene was inserted; WT: wild-type control group. **a** PCR amplification results of the *SAMT* gene in *R. fortunei* (A:DL 2000 Marker; B, C:*RfSAMT*; D-K: Not covered in this article) **b** Relative expression of *SAMT* under different treatments, **c** Relative content of methyl salicylate under different treatments

The key gene *SAMT*, which regulates methyl salicylate synthesis, was first discovered in 1999 [37]. *SAMT* uses S-adenosyl-l-methionine (SAM) and SA as substrates to synthesize methyl salicylate. *SAMT* has strong substrate specificity, and the pathway by which generate methyl salicylate is synthesized is part of the benzene/phenylpropanoid synthesis pathway; different substrates can be catalysed through the activity of enzymes in this pathway, and different products can be obtained. When the substrate is benzoic acid, SA can be formed under the catalytic activity of benzoic acid 2-hydroxylase (*BA2H*), and under the action of benzoic acid carboxyl methyl transferase (*BAMT*), the volatile methyl benzoate can be directly generated. *RfSAMT* expression was found to be significant negatively correlated with methyl salicylate content. It is speculated that when the expression of *SAMT* increases, the content of methyl salicylate increases, while the content of methyl salicylate decreases. In this study, the content of methyl salicylate decreased after transient infection of YJ compared with WT YJ. After transient overexpression, the content of methyl salicylate was 0.17 times that in the WT (Fig. 10b), and the content of methyl benzoate was 1.24 times that in the WT (Fig. 10b). The experimental results were consistent with the predicted results and Th showed that *RfSAMT* expression was significantly negatively correlated with methyl salicylate content. The relative contents of linalool, methyl benzoate, α-terpineol, 3-carene, and other metabolites increased compared with those in WT *R. fortunei* petals. Donghong et al. [42] cloned *CpSAMT* from Chinese plum and transformed it into tobacco to obtain transgenic tobacco plants; the authors found that the floral components of tobacco changed, and methyl salicylate and methyl benzoate could not be detected in the transgenic tobacco. However, large amounts of hexenal, benzyl alcohol, and linalool were detected, which was in complete contrast to WT tobacco plants. These findings are consistent with the findings of the present study. In plants, methyl salicylate is synthesized only from SA [42], and in plants, SA can be synthesized through two distinct pathways. In the the cinnamic acid pathway, phenylalanine undergoes a series of reactions to form SA [43]. Gomez et al. [44] found that methyl salicylate contents increased approximately sevenfold when exogenous SA was applied. After *RfSAMT* was overexpressed, there may have been rapid consumption of SA in YJ without exogenous SA, thereby affecting the metabolism of its upstream metabolites and the original metabolite synthesis pathway and thus leading to the accumulation of increased amounts of linalool, methyl benzoate, alpha-terpineol, and 3-carene. This indicated that overexpression of not every transgene can increase the content of their corresponding metabolites and that these transgenes may damage or interfere with the genes at the insertion point, reducing the content of corresponding metabolites. Moreover, gene expression was negatively correlated with metabolite accumulation.

## Conclusions

In summary, using transcriptomic and metabolomic data and screening DEGs and differentially accumulated metabolites for a correlation analysis, we found nine candidate genes involved in the regulation of the phenylpropanoid pathway in YJ. The expression patterns of nine candidate genes in the petals of the two rhododendron varieties at different stages were further determined. The expression of all these genes was upregulated in YJ but barely detectable in NW. Finally, the function of the *RfSAMT* gene was verified, and it was proven that *SAMT* regulates the biosynthesis of aromatic compounds in YJ. These results provide new insights into the study of the accumulation of floral aromatic compounds and provide valuable reference data for future research on the physiological process and molecular mechanism of the synthesis of benzene/phenylpropanoid-like floral compounds.

## Methods

### Plant materials and sampling

The test materials used were 9-year-old *R. fortunei* (YJ) and 15-year-old *Rhododendron* ‘Nova Zembla’ (NW) plants, which were growing in Siming Mountain National Forest Park, Ningbo city, Zhejiang Province. Voucher specimens of these rhododendron varieties are deposited in the germplasm resource garden of Siming Mountain National Forest Park, Ningbo city, Zhejiang Province, and public sampling is allowed there. The formal identification of the species was conducted by the corresponding author of this article. Ten YJ plants displaying with similar growth and 10 NW plants displaying with similar growth were randomly selected, and flower buds at different stages of opening were harvested without causing mechanical damage. Then, they were grouped into four developmental stages according to the degree of petal opening: the bud stage (petals are tightly closed), first-bloom stage (petals are not fully opened), bloom stage (petals are fully open), and decay stage (withered petals). The stamens were removed, after which the petals were quickly frozen in liquid nitrogen and then stored at − 80 °C for further analysis. The four samples of YJ and NW used for the metabolomic analysis were designated NW1–4 and YJ1–4, respectively, and six biological replicates were included per sample; those used for the RNA-seq analysis were designated NWA-D and YJA-D, respectively, and three biological replicates were included for sample.

### Metabolite detection via GC–TOF–MS and LC–MS

By applying GC–TOF–MS and LC–MS techniques, we detected metabolites in the two *Rhododendron* varieties (NW and YJ) at different developmental stages. A total of 100 ± 10 mg of each sample was placed into a 20 Ml headspace vial, and 10 Μl of 2-octanol (as an internal standard) was added. The volatile metabolites in the petal samples were extracted by an SPME-PAL gas-phase solid-phase microextraction automatic sampling system for subsequent GC–TOF–MS detection and analysis. GC–TOF–MS analysis was performed using an Agilent 7890 gas chromatograph system coupled to a 5977B mass spectrometer; the system utilized DB-Wax. LC–MS was used to detect nonvolatile metabolites in the samples. An appropriate amount of sample was ground in liquid nitrogen, 50 mg of ground sample was added to an Eppendorf tube, and 1000 Μl of extraction solution (methanol:water = 3:1, with isotopically labelled internal standard mixture) was added. Then, the samples were homogenized at 35 Hz for 4 min and sonicated for 5 min in an ice water bath; the homogenization and sonication cycles were repeated three times. Then, the samples were incubated for 1 h at − 40 °C and centrifuged at 12000 rpm for 15 min at 4 °C. The resulting supernatant was transferred to a fresh glass vial for analysis. A quality control (QC) sample was prepared by mixing an equal aliquot of the supernatants from all of the samples. LC–MS analyses were performed using an UHPLC system (Thermo Fisher Scientific, MA, USA) equipped with a UPLC HSS T3 column (2.1 mm × 100 mm, 1.8 μm) coupled to a Q Exactive HFX mass spectrometer (Thermo Fisher Scientific, MA, USA). The mobile phase consisted of 5 mmol/L ammonium acetate and 5 mmol/L acetic acid in water (A) and acetonitrile (B). The autosampler temperature was 4 °C, and the injection volume was 3 Μl. Statistical significance and fold-changes of the abundance of metabolites between samples were tested by Student’s *t* test. Metabolites for whose contents differed according to a *P* value ≤0.05 and a VIP ≥ 1 were considered significantly differentially accumulated.

### Metabolomic data analysis

First, a QC analysis was performed to verify the reliability of the data. The data detected by GC–TOF–MS were analysed using Chroma TOF 4.3X software from LECO Corporation (San Jose, CA, USA) and via information from the Nist database for raw peak extraction, filtering of data baselines, calibration of the baseline, peak alignment, deconvolution analysis, peak identification, integration and spectrum matching of the peak area. The raw data detected by LC–MS were converted to mzXML format using ProteoWizard and processed with a program written by staff at the biomedical company Shanghai Yuanxin Biomedical Technology Co., Ltd. (Shanghai, China). The program was developed using R and is based on XCMS for peak detection, extraction, alignment, and integration. Then, an in-house MS2 database (BiotreeDB) was applied for metabolite annotation. The raw data were analysed as described by Dunn et al. [45].

### RNA extraction, library construction, and sequencing

Total RNA was extracted from eight groups of samples using a TRIzol reagent kit (Invitrogen, Carlsbad, CA, USA) according to the manufacturer’s instructions, and three biological replicates were included. Total RNA sample QC, library construction, and sequencing were conducted by staff at BGI Tech (Shenzhen, China). The RNA concentration and quality were tested using an Agilent 2100 Bioanalyzer (Agilent RNA 6000 Nano Kit) (Agilent, CA, USA), which included determining the RNA concentration, RNA integrity number (RIN) value, 28S/18S, and fragment length distribution. The Mrna was enriched by Oligo (Dt) magnetic beads, and then the obtained RNA was fragmented with interrupt buffer. First-strand complementary DNA (Cdna) was synthesized by N6 random primers, and double-stranded cDNAs were prepared by the use of these short fragments as templates. Adapters were ligated to the short fragments using T4 DNA ligase (Invitrogen, Shanghai, China), and after end repair and ligation of the adapters, the products were enriched by PCR to generate Cdna libraries. The libraries were subjected to sequencing with the sequencing platform DNBSEQ (BGI, Shenzhen, China), and the products were considered raw reads.

### Transcriptomic data analysis

All the generated raw sequencing reads were filtered to remove the reads containing adapter sequences, low-quality reads, and reads comprising more than 5% ambiguous residues (N) by SOAPnuke software (BGI, Shenzhen, China). After filtration, the remaining reads were considered clean reads and stored in FASTQ format. Trinity software was used to de novo assemble the clean reads, and then TGICL was used to cluster the assembled transcripts to remove redundant reads to obtain unigenes. TGICL was used again to cluster the eight samples of unigenes to remove any redundant sequences and obtain the final unigenes for follow-up analysis. All the assembled nonredundant and filtered unigenes were annotated by BLAST software against the contents within the Nr database, the Nt database, the Pfam database, the SwissProt protein database, the KOG database, the GO database and the KEGG database [46–48]. The relative levels of transcripts were calculated using Bowtie2 [49] and RSEM [50], and the relative expression levels were normalized using fragments per kilobase per million mapped reads (FPKM) values. DEGs were identified based on a negative binomial distribution model using DEseq2 software [51], and the screening criterion was a Q value (adjusted *P* value) ≤ 0.05.

### qRT–PCR validation

Total RNA was extracted with an RNAprep Pure Plant Plus Kit (TIANGEN, Beijing, China), and cDNA was synthesized using a NovoScript Plus All-in-One 1st Strand cDNA Synthesis SuperMix (gDNA Purge) Reverse Transcription Kit (Novoprotein, Shanghai, China). Primer 6 software was used to design specific primers for nine genes identified from the transcriptomic data (Additional file 2: Table S5). EF1α was used as an internal reference gene. A TransStart Tip Green qPCR Super rMixS Kit (TransGen Biotech, Beijing, China) was used for qRT–PCR, and each reaction was replicated three times. The 2^-ΔΔCT^ method was used for data analysis, SPSS 26.0 was used for one-way ANOVA, and Origin software was used for constructing the figures.

### *RfSAMT* gene functional verification

#### Cloning of *RfSAMT* and constructing pCAMBIA1302-*SAMT* overexpression vectors

Using YJ petals as experimental material, we extracted total RNA from the samples using a TRIzol reagent kit (Invitrogen, Carlsbad, CA, USA) following the manufacturer’s instructions, and the total RNA was reverse transcribed using a NovoScript Plus All-in-One 1st Strand cDNA Synthesis SuperMix (gDNA Purge) Reverse Transcription Kit (Novoprotein, China) to synthesize first-strand cDNA. With cDNA as a template and the designed primer Q-SAMT (Additional file 2: Table S6), RT–PCR was used to amplify the full-length sequence of *RfSAMT*. The PCR product was purified via an agarose gel recovery kit and then inserted and ligated into a pEASY-Blunt Zero cloning vector for transformation into DH5α competent cells and selected by colony PCR. Then, the bacterial solution was sent to Beijing Qingke Biotechnology Co., Ltd. (Beijing, China), for sequencing verification. The bacterial solution with correct sequence was shaken, and the plasmids were extracted with an E.Z.N.A.® HP Plant DNA Kit (Shanghai Solarbio Bioscience and Technology Co. Ltd., Shanghai, China) according to the manufacturer’s instructions. The pCAMBIA1302 vector was linearized with Ncol-HF restriction endonuclease, and the primer G-SAMT (Additional file 2: Table S6) was designed and used for PCR amplification. After the *SAMT* target gene was inserted and ligated into the vector, DH5α competent cells were transformed. After screening positive clones with YJ primers (Additional file 2: Table S6), we sent the bacterial solution to Beijing Qingke Biotechnology Co., Ltd., for sequencing verification. The plasmid was subsequently extracted from the bacterial liquid with the correct sequencing result, and the recombinant plasmid was transformed into Agrobacterium GV3101 competent cells via the freeze–thaw method. The selected colonies were then verified by bacterial liquid PCR in conjunction with YJ primers, and pCAMBIA1302-*SAMT* recombinant plasmids were obtained. The bacterial liquid in which the positive transformants were detected was stored in 50% glycerol at a ratio of 1:1 at − 80 °C for later use.

#### Agrobacterium-mediated transient infection of *R. fortunei*

After successful sequencing, samples were picked and added to 3 mL of Luria–Bertani (LB) liquid media consisting of 50 mg/L kanamycin and 50 mg/L rifampin, after which the solution was shaken at 200 r/min for 12 h at 28 °C. Then, the bacterial solution was shaken under the same culture conditions at a ratio of 1:50 between the bacterial solution and the LB liquid medium containing antibiotics. The supernatant was subsequently removed by centrifugation at 5000 rpm, and the bacterial cells were resuspended in buffer (MgCl_2_, 10 mM; acetosyringone, 0.1 mM; 2-(N-morpholine) ethanesulfonic acid, 10 mM; pH 5.8). The OD 600 value was adjusted to 0.8 to obtain a sufficient Agrobacterium infection solution. Twenty unopened flower buds were randomly selected for injection with the infection solution. An additional 20 unopened flower buds displaying the same growth status were selected at the same time and injected with an Agrobacterium infection solution with the empty pCAMBIA1302 plasmid without the *SAMT* target gene. The solutions were incubated in the dark for 3 days in an opaque protective bag. Then, the bag was removed, and the solutions were cultured under natural conditions.

#### Changes in gene expression and contents of floral aromatic compounds and related compounds after overexpression of *RfSAMT*

The petals and WT petals injected with the Agrobacterium infection solution with the empty pCAMBIA1302 vector and the Agrobacterium infection solution with the recombinant pCAMBIA1302-*SAMT* vector were used as materials for RNA extraction; three biological replicates were included per each group. By using the qRT–PCR verification method, we detected changes in *RfSAMT* gene expression. 2-Octanol was used as a standard according to the methods of Yao Chenyang et al. [52], with modifications. HS-SPME-GC–MS was used to determine the composition and relative contents of volatile metabolites in the different samples.

## Supplementary Information

**Additional file 1: Figure S1.** Volcano plot of NW2vsYJ2 differential metabolites detected by GC-TOFMS. **Figure S2.** Volcano plot of NW3vsYJ3 differential metabolites detected by GC-TOFMS. **Figure S3.** Volcano plot of NW4vsYJ4 differential metabolites detected by GC-TOFMS. **Figure S4.** Volcano plot of NW2vsYJ2 differential metabolites detected by LC-MS in POS mode. **Figure S5.** Volcano plot of NW3vsYJ3 differential metabolites detected by LC-MS in POS mode. **Figure S6.** Volcano plot of NW4vsYJ4 differential metabolites detected by LC-MS in POS mode. **Figure S7.** Volcano plot of NW2vsYJ2 differential metabolites detected by LC-MS in NEG mode. **Figure S8.** Volcano plot of NW3vsYJ3 differential metabolites detected by LC-MS in NEG mode. **Figure S9.** Volcano plot of NW4vsYJ4 differential metabolites detected by LC-MS in NEG mode. **Figure S10.** Volcano plot of YJ2vsYJ3 differential metabolites detected by GC-TOFMS. **Figure S11.** Volcano plot of YJ2vsYJ3 differential metabolites detected by LC-MS in POS mode. **Figure S12.** Volcano plot of YJ2vsYJ3 differential metabolites detected by LC-MS in NEG mode. **Figure S13.** Volcano plot of YJ3vsYJ4 differential metabolites detected by GC-TOFMS. **Figure S14.** Volcano plot of YJ3vsYJ4 differential metabolites detected by LC-MS in POS mode. **Figure S15.** Volcano plot of YJ3vsYJ4 differential metabolites detected by LC-MS in NEG mode. **Figure S16.** RNA-seq data analysis. CDS length distribution map. **Figure S17.** Principal component analysis of transcriptomes of different stages (A, B,C, and D) at different varieties (NW and YJ). **Figure S18.** Top 20 KEGG pathways with the most significant DEG enrichment in NWAvsYJA. **Figure S19.** Top 20 KEGG pathways with the most significant DEG enrichment in NWBvsYJB. **Figure S20.** Top 20 KEGG pathways with the most significant DEG enrichment in NWCvsYJC. **Figure S21.** Top 20 KEGG pathways with the most significant DEG enrichment in YJAvsYCB. **Figure S22. **Correlation analysis of NW2 vs. YJ2 phenylpropanoid pathway candidate genes and benzene/phenylpropanoid substances. a. GC-TOFMS, b. LC-MS (POS), c. LC-MS (NEG). **Figure S23. **Correlation analysis of NW3 vs. YJ3 phenylpropanoid pathway candidate genes and benzene/phenylpropanoid substances. a. GC-TOFMS, b. LC-MS (POS), c. LC-MS (NEG). **Figure S24** Correlation analysis of NW4 vs. YJ4 phenylpropanoid pathway candidate genes and benzene/phenylpropanoid substances. a. GC-TOFMS, b. LC-MS (POS), c. LC-MS (NEG). **Figure S25. **Correlation analysis of YJ1 vs. YJ2 phenylpropanoid pathway candidate genes and benzene/phenylpropanoid substances. a. GC-TOFMS, b. LC-MS (POS), c. LC-MS (NEG). **Figure S26. **Correlation analysis of YJ2 vs. YJ3 phenylpropanoid pathway candidate genes and benzene/phenylpropanoid substances. a. GC-TOFMS, b. LC-MS (POS), c. LC-MS (NEG). **Figure S27.** Correlation analysis of YJ3 vs. YJ4 phenylpropanoid pathway candidate genes and benzene/phenylpropanoid substances. a. GC-TOFMS, b. LC-MS (POS), c. LC-MS (NEG).

**Additional file 2: Table S1-S6.**

## Data Availability

Transcriptome sequencing data are available in the SRA database of National Center for Biotechnology Information under the accession number of PRJNA885230 (https://www.ncbi.nlm.nih.gov/sra/PRJNA885230).

## Abbreviations

PAL: Phenylalanine ammonia lyase
C4H: Cinnamate 4-Hydroxylase
4CL: 4-Coumarate-CoA ligase
SAMT: Salicylic acid carboxyl methyl transferase
CCoAOMT: Caffeoyl-CoA 3-O-methyltransferase
CCR: Cinnamoyl-CoA reductase
CAD: Cinnamyl alcohol dehydrogenase
EGS: Eugenol synthase
BALDH: Benzaldehyde dehydrogenase
VOC: Volatile organic compounds
HS-SPME-GC-MS: Headspace solid-phase microextraction combined with gas chromatography-mass spectrometry
GC-TOFMS: Gas chromatography tandem time-of-flight mass spectrometry
LC-MS: Liquid chromatography-mass spectrometry
QC: Quality control
VIP: Variable importance in the projection
PCA: Principal component analysis
WT: Wild type
NCBI: National center for biotechnology information
NR: Non-redundant protein sequence database
NT: Nucleotide sequence database
KOG: Eukaryotic orthologous groups
GO: Gene ontology
KEGG: Kyoto encyclopedia of genes and genome
FPKM: Fragments per kilobase per million mapped reads
qRT-PCR: Quantitative reverse-transcription PCR
PC1: First principal component
SA: Salicylic acid PC1: First principal component
PC2: Second principal component
