# Supplementary material for: Transcriptomic and metabolomic data reveal key genes that are involved in the phenylpropanoid pathway and regulate the floral fragrance of Rhododendron fortunei
Source: BMC Plant Biol. 2023 Jan 5;23:8. doi: 10.1186/s12870-022-04016-7 (PMC9814181; doi:10.1186/s12870-022-04016-7)
Supplement: Supplementary file 1 — Additional file 1: Figure S1. Volcano plot of NW2vsYJ2 differential metabolites detected by GC-TOFMS. Figure S2. Volcano plot of NW3vsYJ3 differential metabolites detected by GC-TOFMS. Figure S3. Volcano plot of NW4vsYJ4 differential metabolites detected by GC-TOFMS. Figure S4. Volcano plot of NW2vsYJ2 differential metabolites detected by LC-MS in POS mode. Figure S5. Volcano plot of NW3vsYJ3 differential metabolites detected by LC-MS in POS mode. Figure S6. Volcano plot of NW4vsYJ4 differential metabolites detected by LC-MS in POS mode. Figure S7. Volcano plot of NW2vsYJ2 differential metabolites detected by LC-MS in NEG mode. Figure S8. Volcano plot of NW3vsYJ3 differential metabolites detected by LC-MS in NEG mode. Figure S9. Volcano plot of NW4vsYJ4 differential metabolites detected by LC-MS in NEG mode. Figure S10. Volcano plot of YJ2vsYJ3 differential metabolites detected by GC-TOFMS. Figure S11. Volcano plot of YJ2vsYJ3 differential metabolites detected by LC-MS in POS mode. Figure S12. Volcano plot of YJ2vsYJ3 differential metabolites detected by LC-MS in NEG mode. Figure S13. Volcano plot of YJ3vsYJ4 differential metabolites detected by GC-TOFMS. Figure S14. Volcano plot of YJ3vsYJ4 differential metabolites detected by LC-MS in POS mode. Figure S15. Volcano plot of YJ3vsYJ4 differential metabolites detected by LC-MS in NEG mode. Figure S16. RNA-seq data analysis. CDS length distribution map. Figure S17. Principal component analysis of transcriptomes of different stages (A, B,C, and D) at different varieties (NW and YJ). Figure S18. Top 20 KEGG pathways with the most significant DEG enrichment in NWAvsYJA. Figure S19. Top 20 KEGG pathways with the most significant DEG enrichment in NWBvsYJB. Figure S20. Top 20 KEGG pathways with the most significant DEG enrichment in NWCvsYJC. Figure S21. Top 20 KEGG pathways with the most significant DEG enrichment in YJAvsYCB. Figure S22. Correlation analysis of NW2 vs. YJ2 phenylpropanoid pathway candidate genes and benze [file 12870_2022_4016_MOESM1_ESM.pdf]

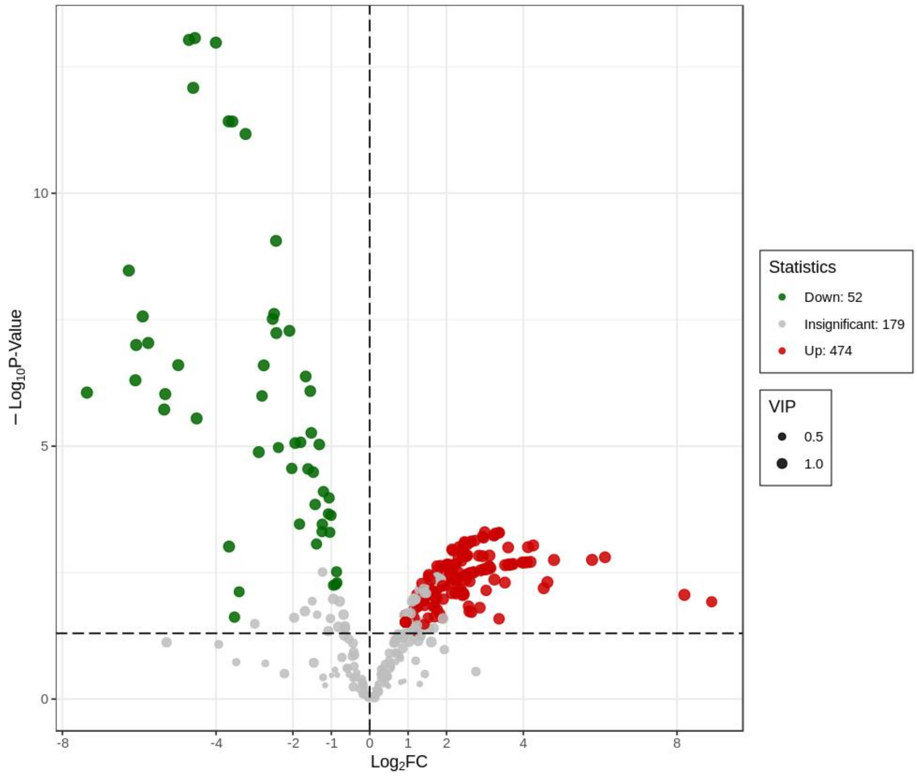

Figure S1. Volcano plot of NW2vsYJ2 differential metabolites detected by GC-TOFMS.

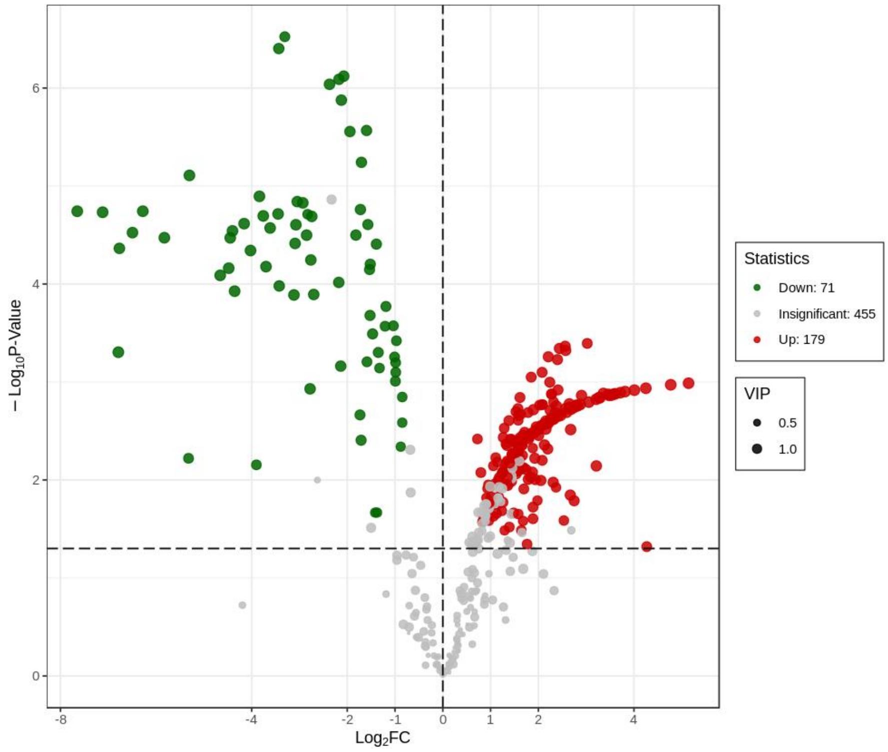

Figure S2. Volcano plot of NW3vsYJ3 differential metabolites detected by GC-TOFMS.

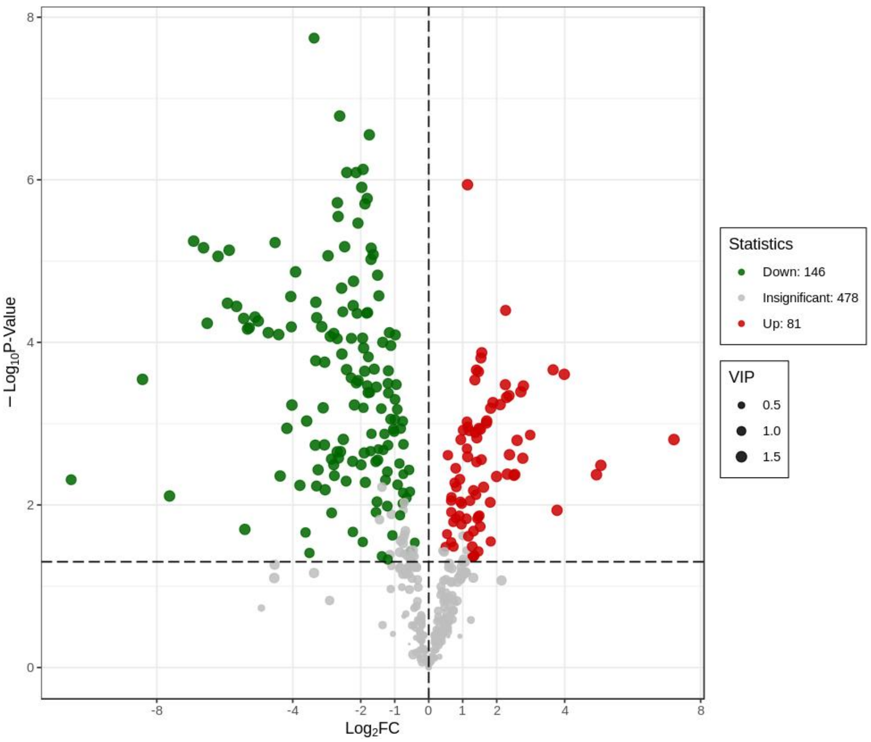

Figure S3. Volcano plot of NW4vsYJ4 differential metabolites detected by GC-TOFMS.

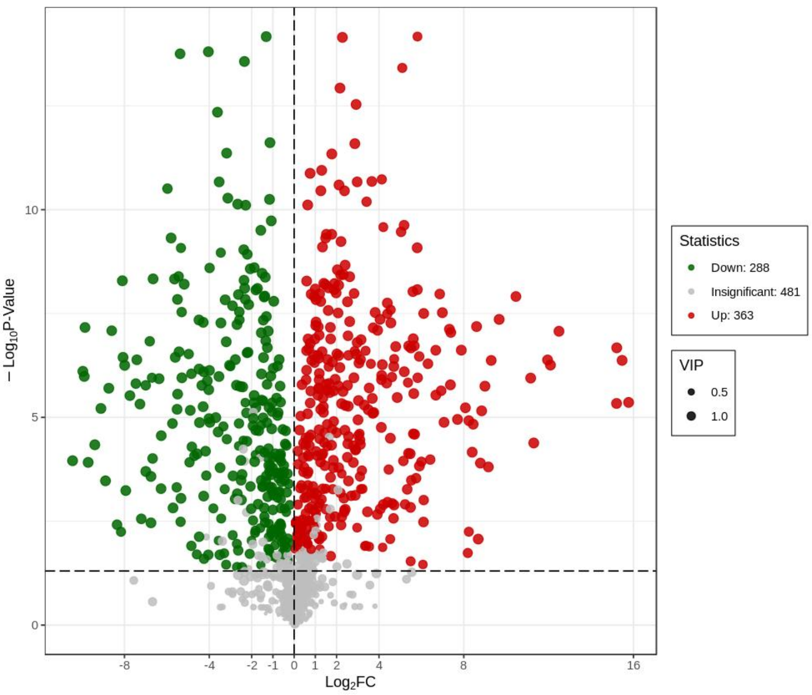

Figure S4. Volcano plot of NW2vsYJ2 differential metabolites detected by LC-MS in POS mode.

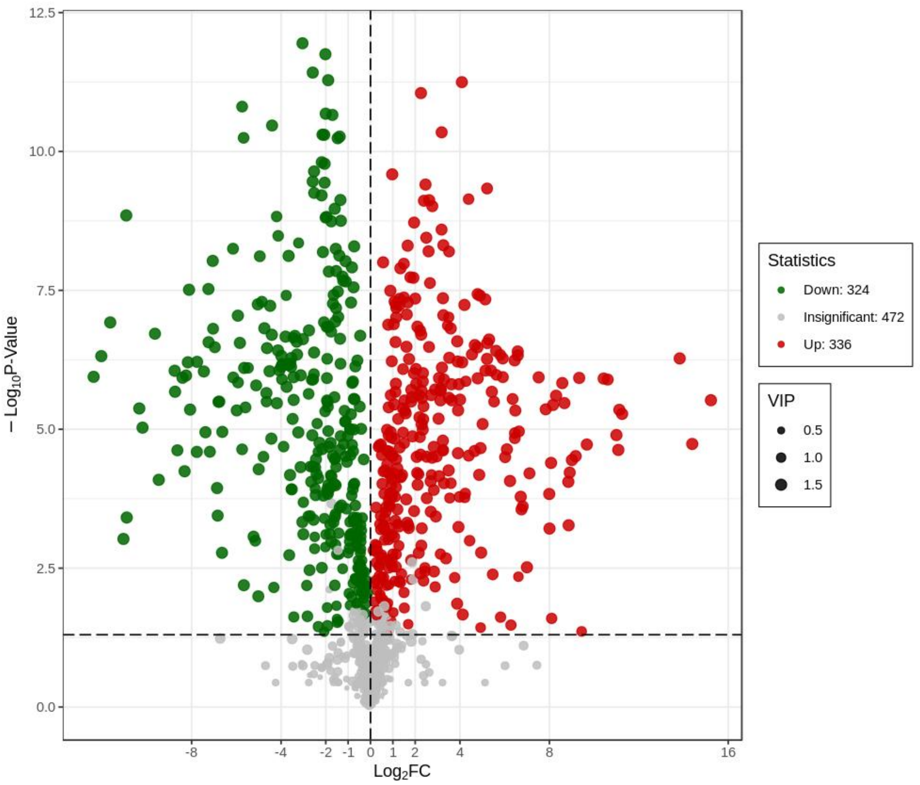

Figure S5. Volcano plot of NW3vsYJ3 differential metabolites detected by LC-MS in POS mode.

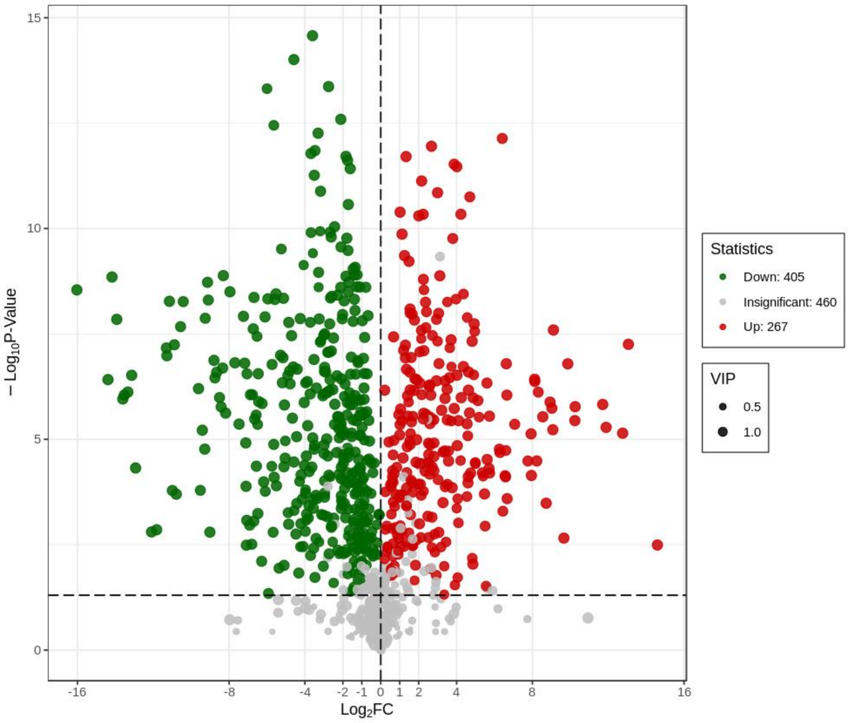

Figure S6. Volcano plot of NW4vsYJ4 differential metabolites detected by LC-MS in POS mode.

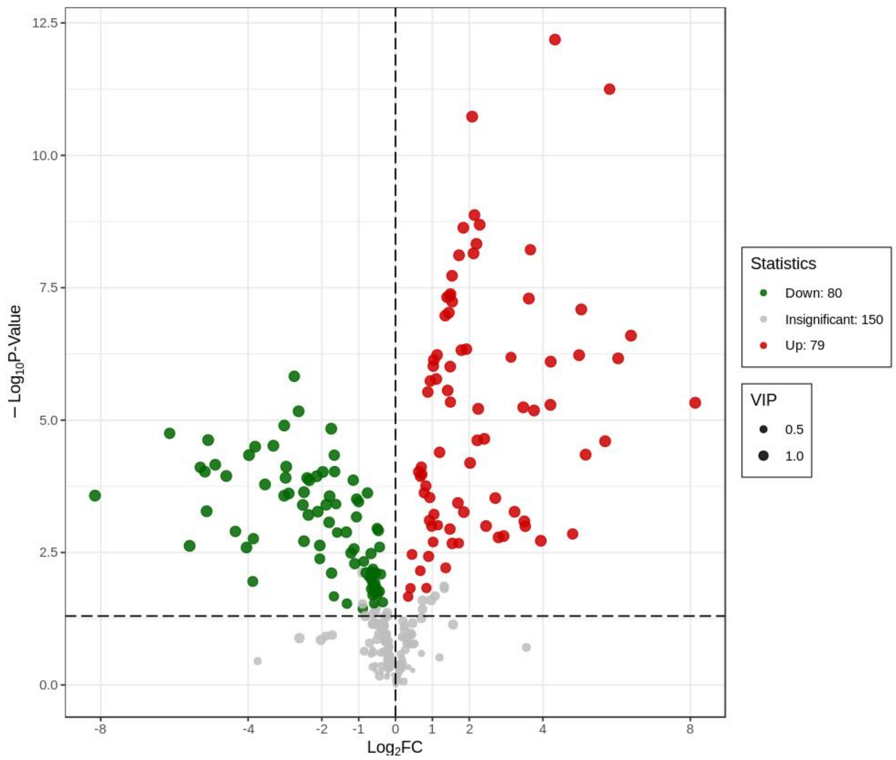

Figure S7. Volcano plot of NW2vsYJ2 differential metabolites detected by LC-MS in NEG mode.

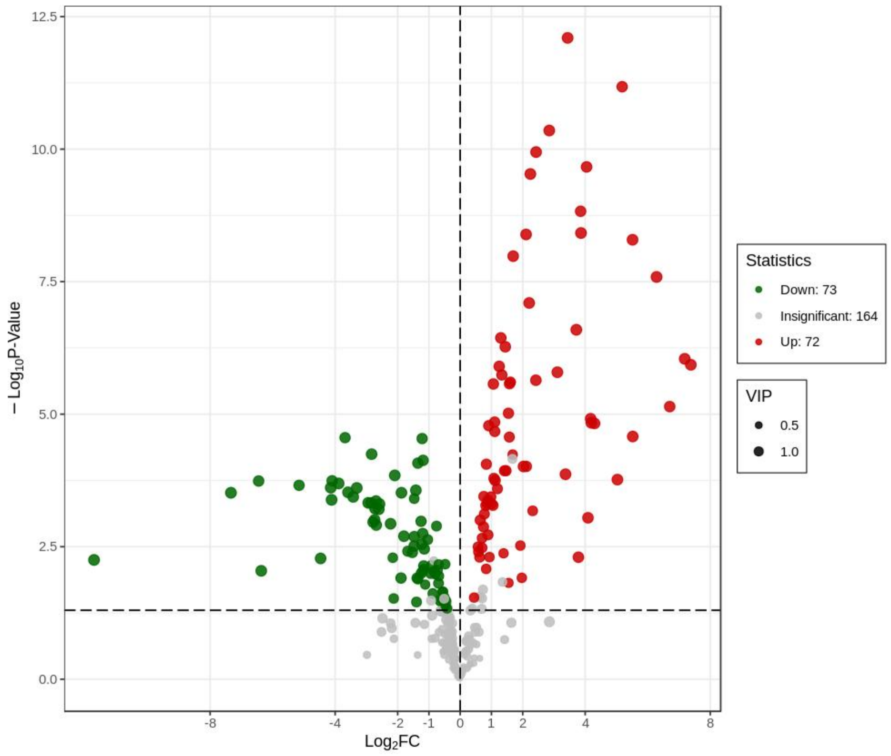

Figure S8. Volcano plot of NW3vsYJ3 differential metabolites detected by LC-MS in NEG mode.

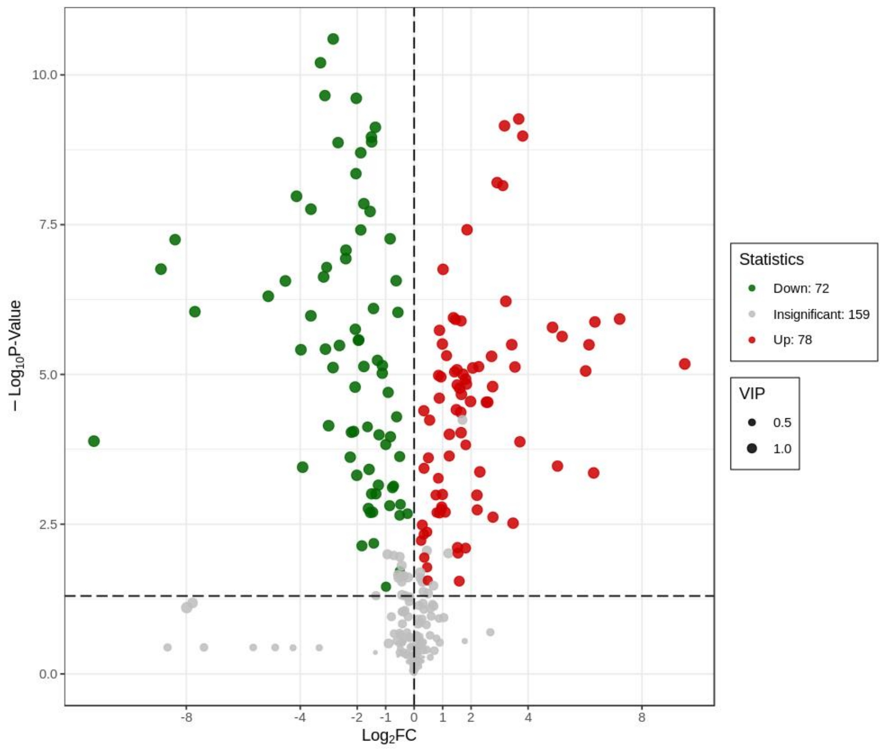

Figure S9. Volcano plot of NW4vsYJ4 differential metabolites detected by LC-MS in NEG mode.

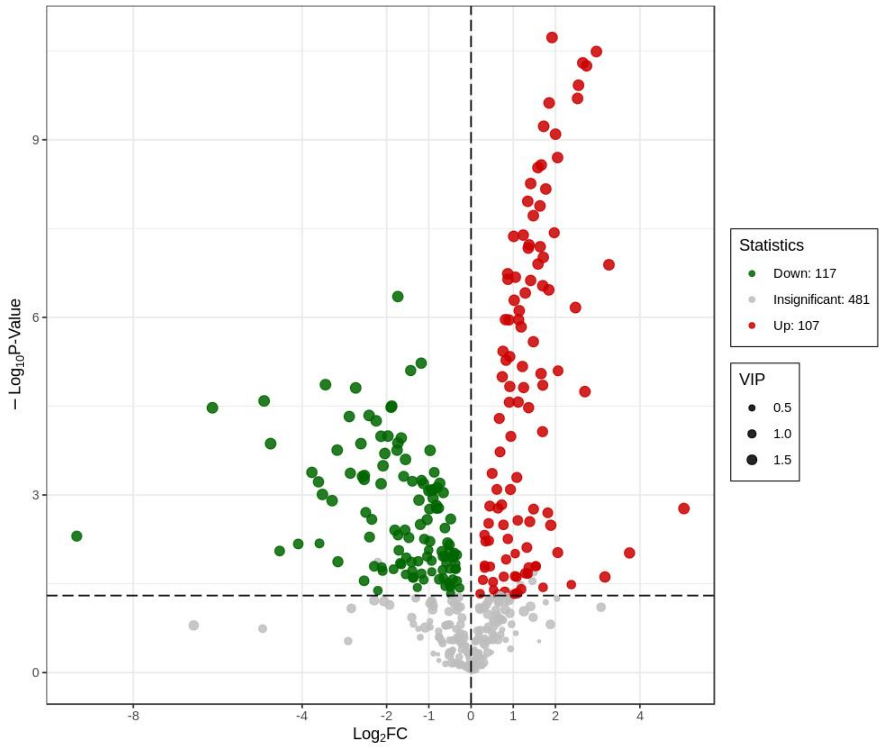

Figure S10. Volcano plot of YJ2vsYJ3 differential metabolites detected by GC-TOFMS.

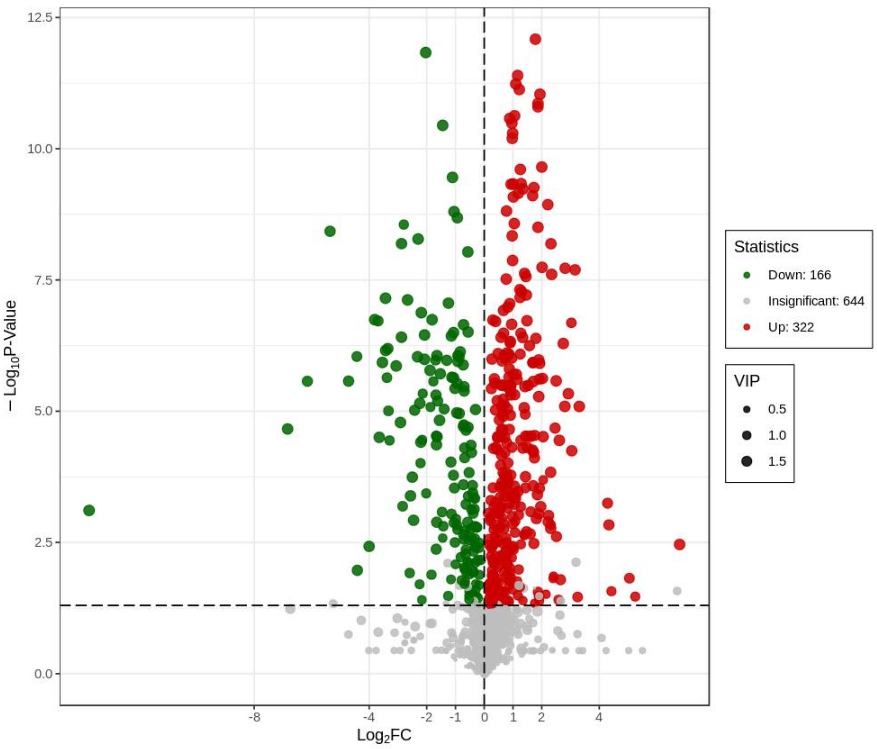

Figure S11. Volcano plot of YJ2vsYJ3 differential metabolites detected by LC-MS in POS mode.

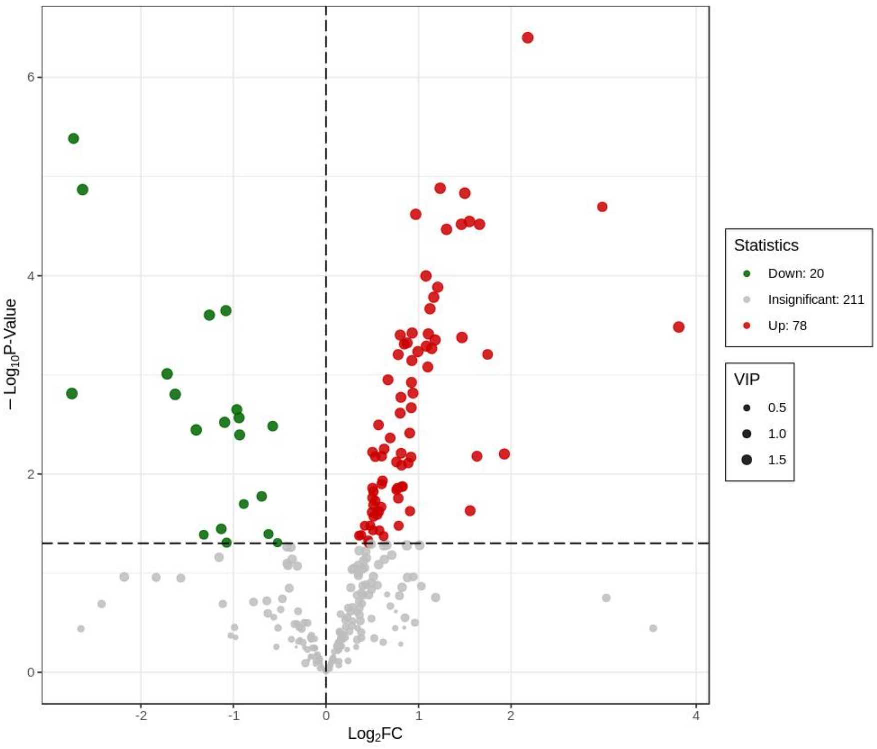

Figure S12. Volcano plot of YJ2vsYJ3 differential metabolites detected by LC-MS in NEG mode.

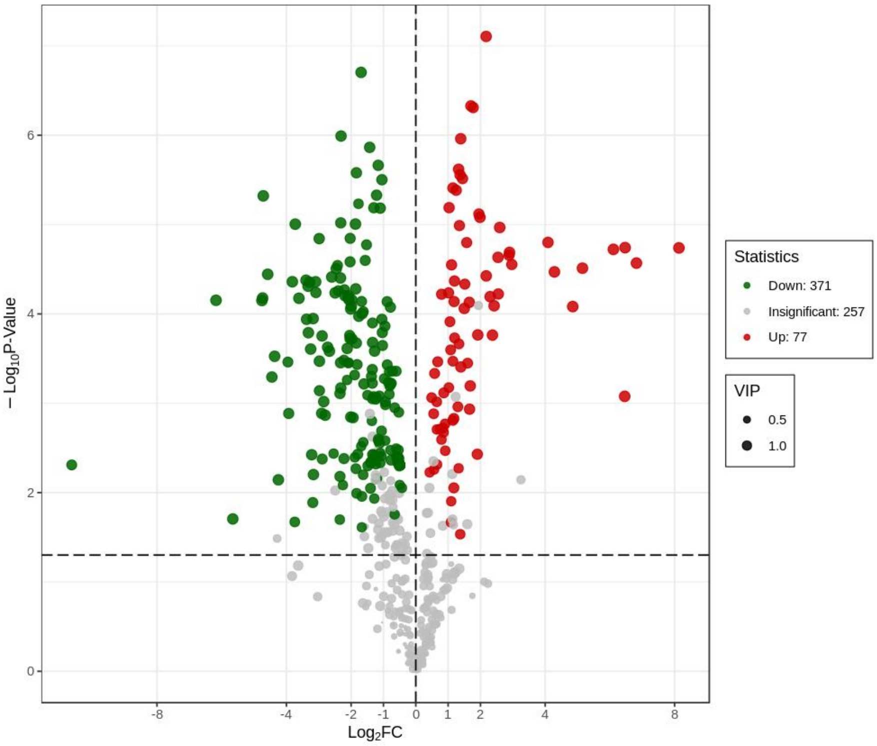

Figure S13. Volcano plot of YJ3vsYJ4 differential metabolites detected by GC-TOFMS.

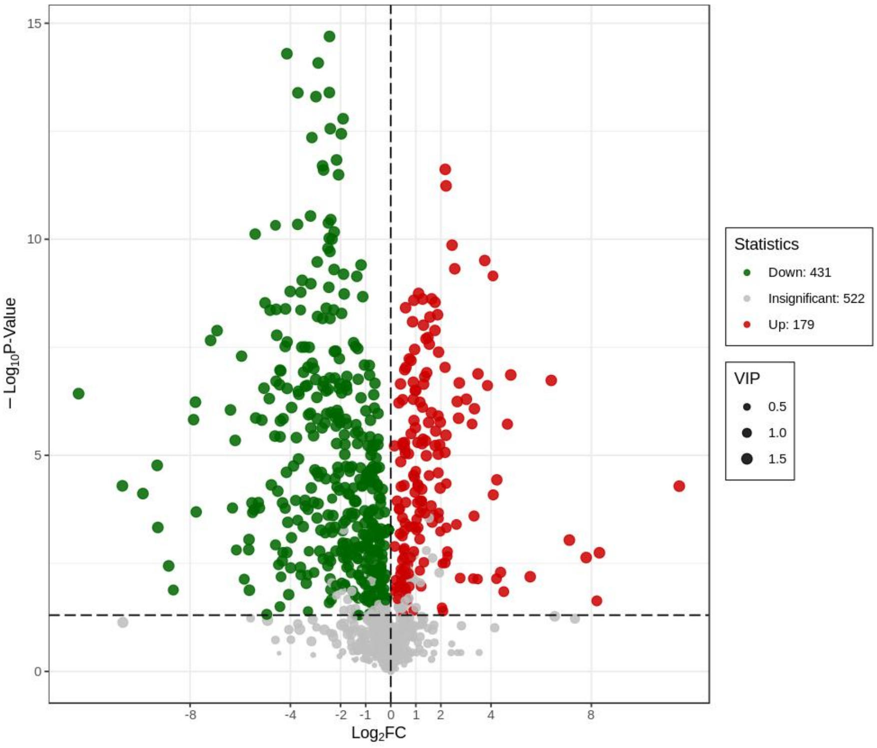

Figure S14. Volcano plot of YJ3vsYJ4 differential metabolites detected by LC-MS in POS mode.

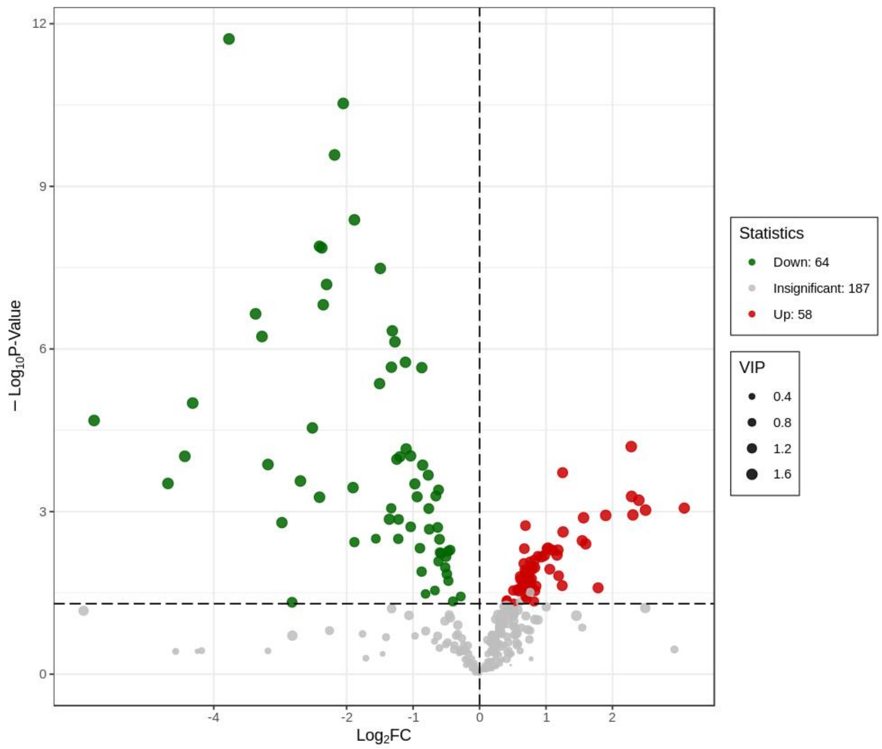

Figure S15. Volcano plot of YJ3vsYJ4 differential metabolites detected by LC-MS in NEG mode.

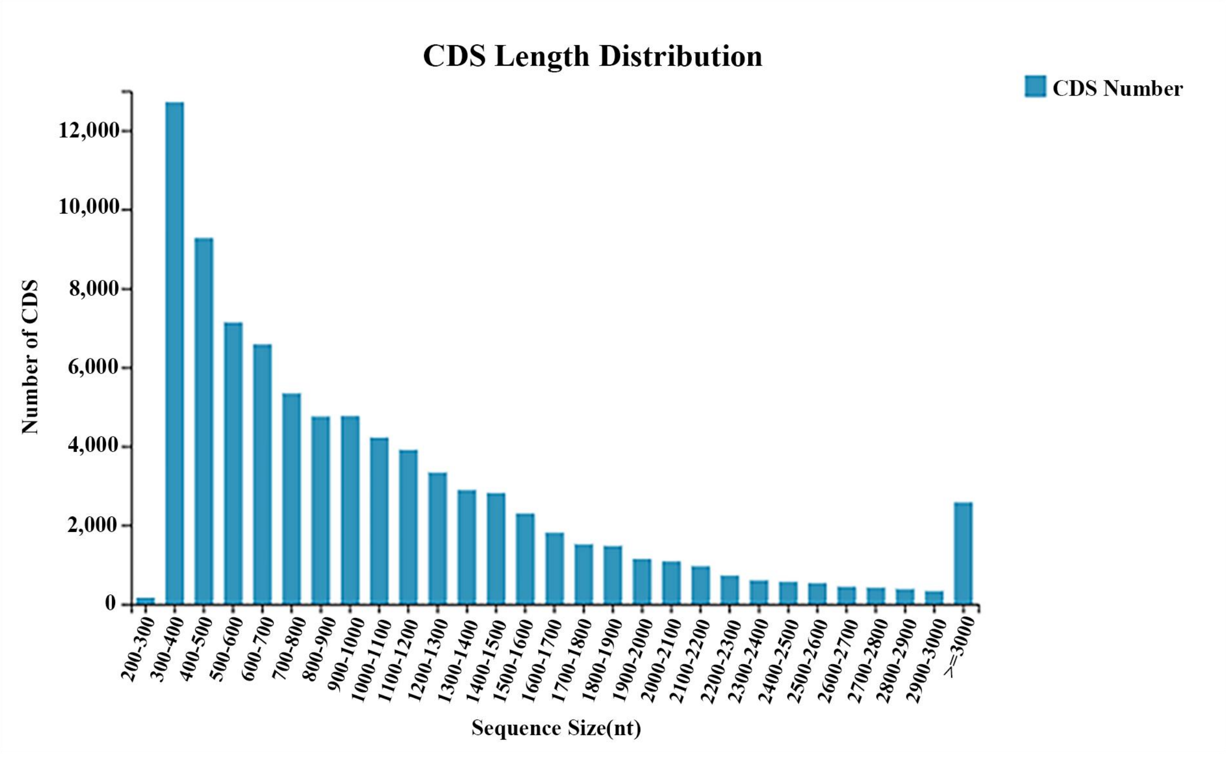

Figure S16. RNA-seq data analysis. CDS length distribution map.

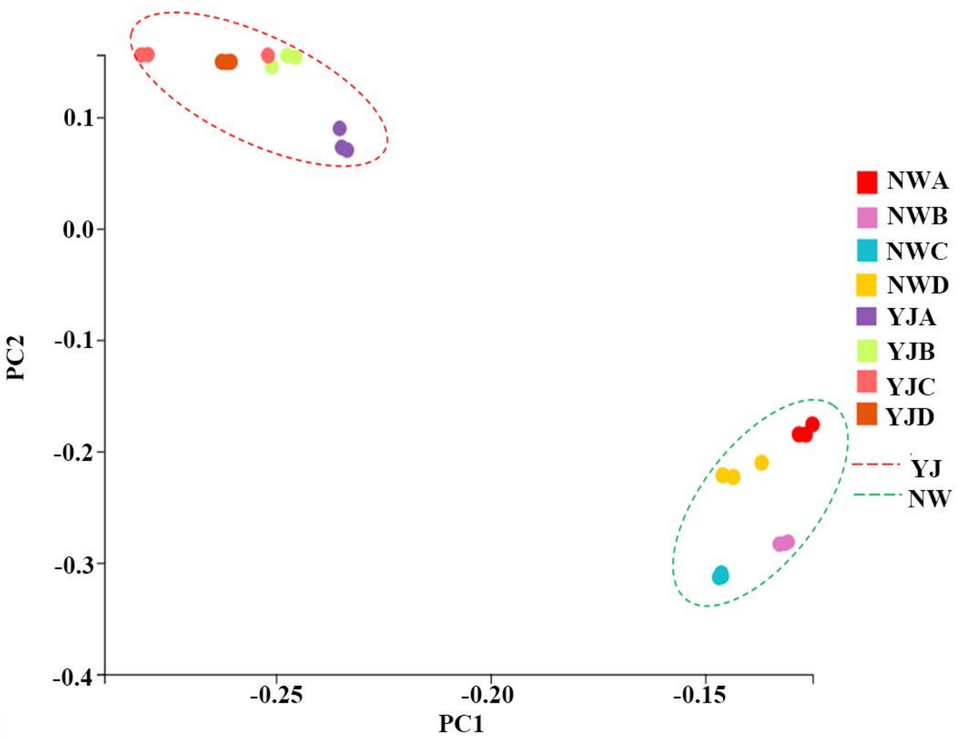

Figure S17. Principal component analysis of transcriptomes of different stages (A, B, C, and D) at different varieties (NW and YJ).

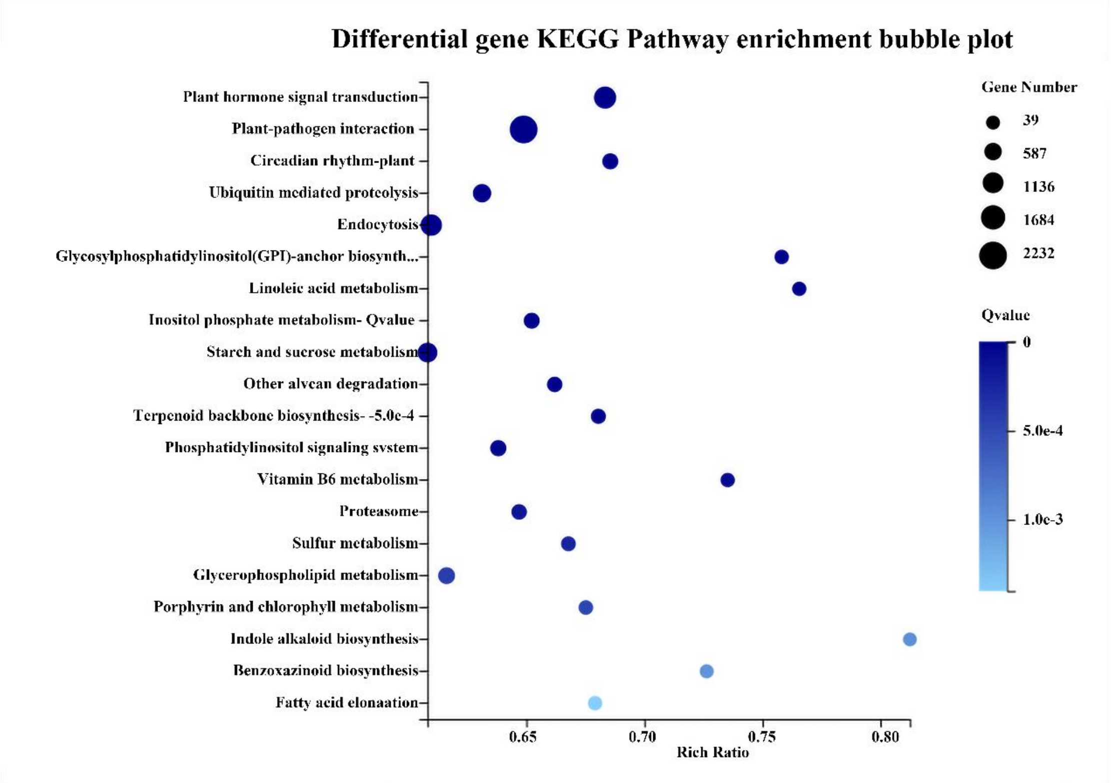

Figure S18. Top 20 KEGG pathways with the most significant DEG enrichment in NWAvsYJA.

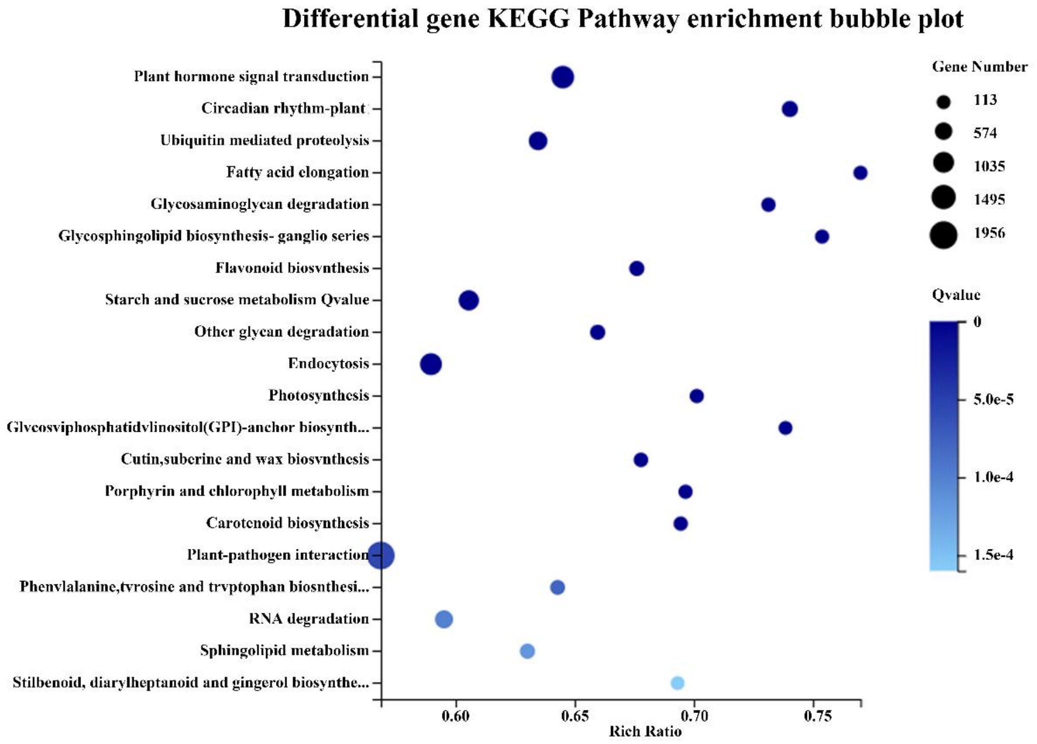

Figure S19. Top 20 KEGG pathways with the most significant DEG enrichment in NWBvsYJB.

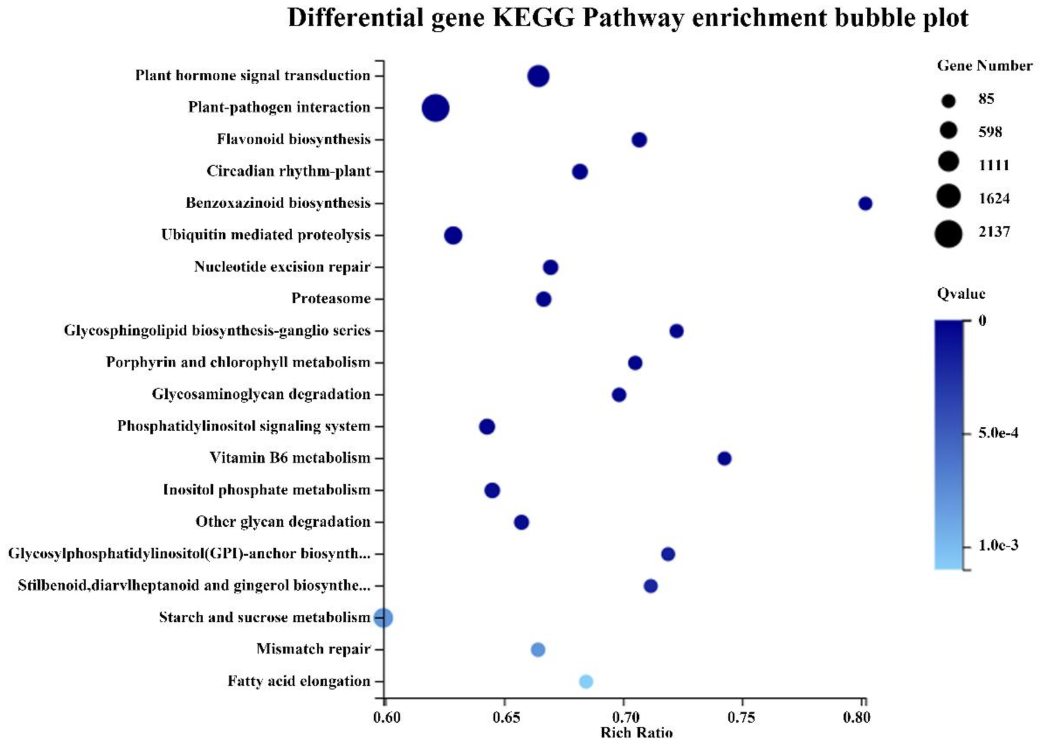

Figure S20. Top 20 KEGG pathways with the most significant DEG enrichment in NWCvsYJC.

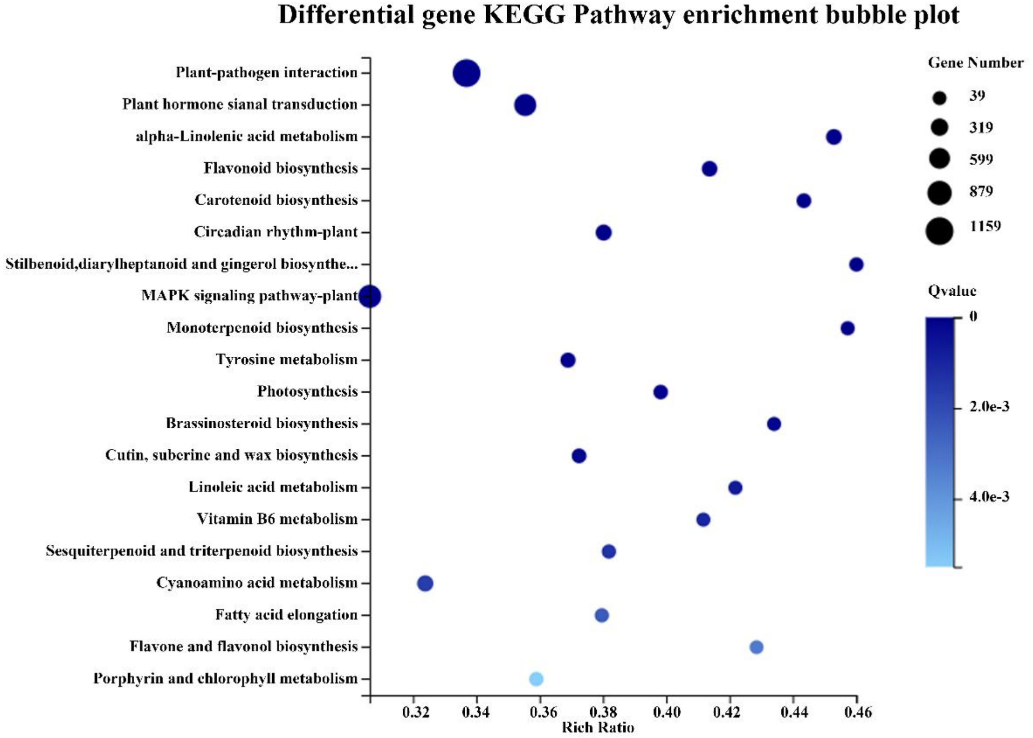

Figure S21. Top 20 KEGG pathways with the most significant DEG enrichment in YJAvsYCB.

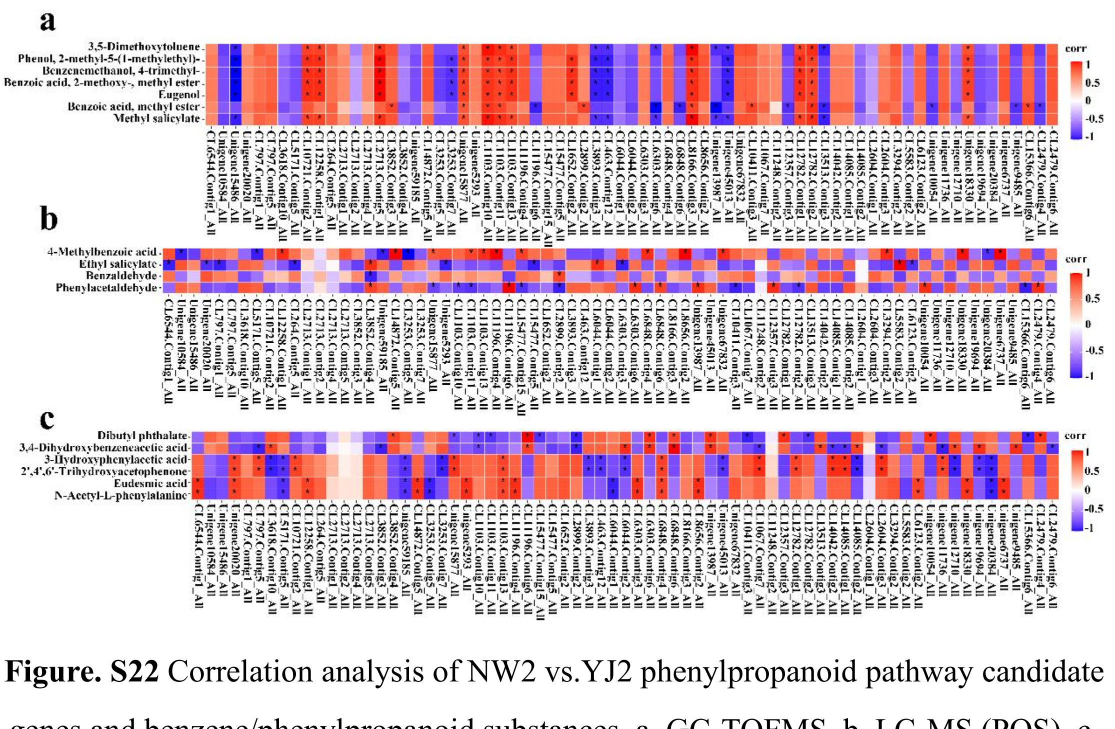

Figure. S22 Correlation analysis of NW2 vs.YJ2 phenylpropanoid pathway candidate genes and benzene/phenylpropanoid substances. a. GC-TOFMS, b. LC-MS (POS), c. LC-MS (NEG).

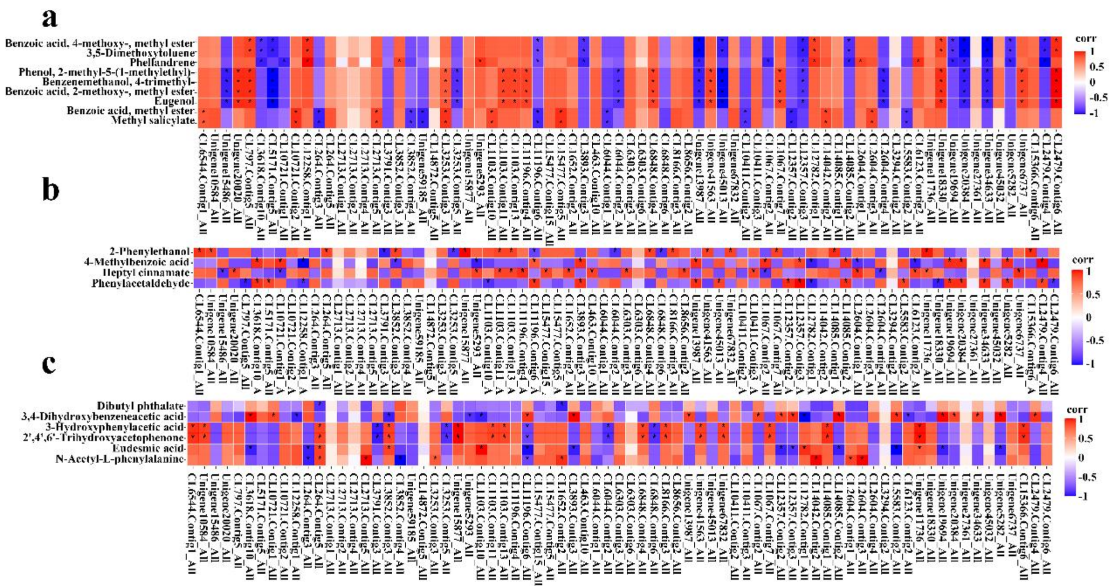

Figure. S23 Correlation analysis of NW3 vs.YJ3 phenylpropanoid pathway candidate genes and benzene/phenylpropanoid substances. a. GC-TOFMS, b. LC-MS (POS), c. LC-MS (NEG).

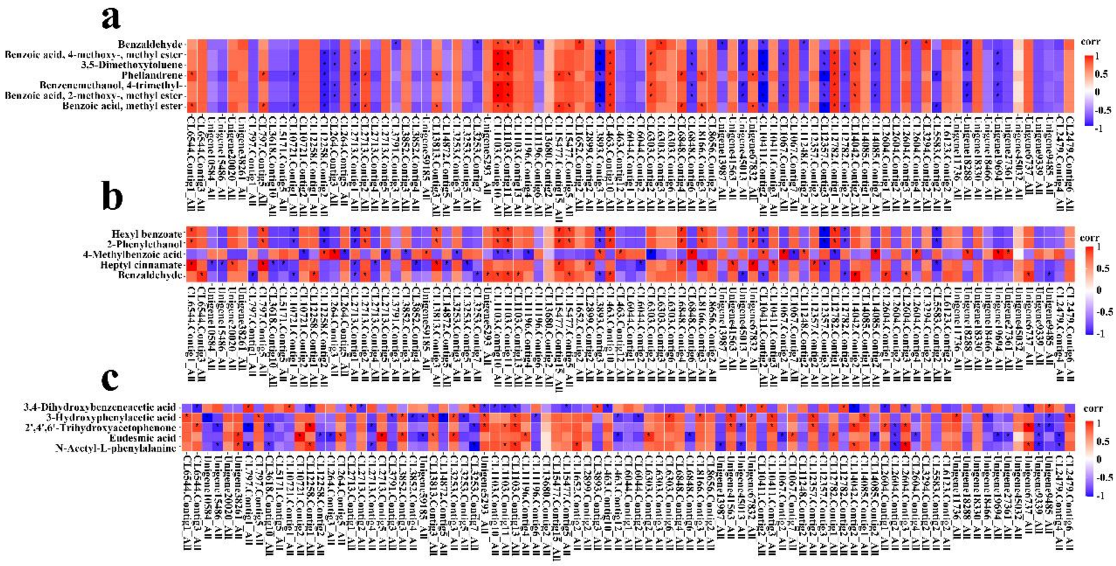

Figure. S24 Correlation analysis of NW4 vs.YJ4 phenylpropanoid pathway candidate genes and benzene/phenylpropanoid substances. a. GC-TOFMS, b. LC-MS (POS), c. LC-MS (NEG).

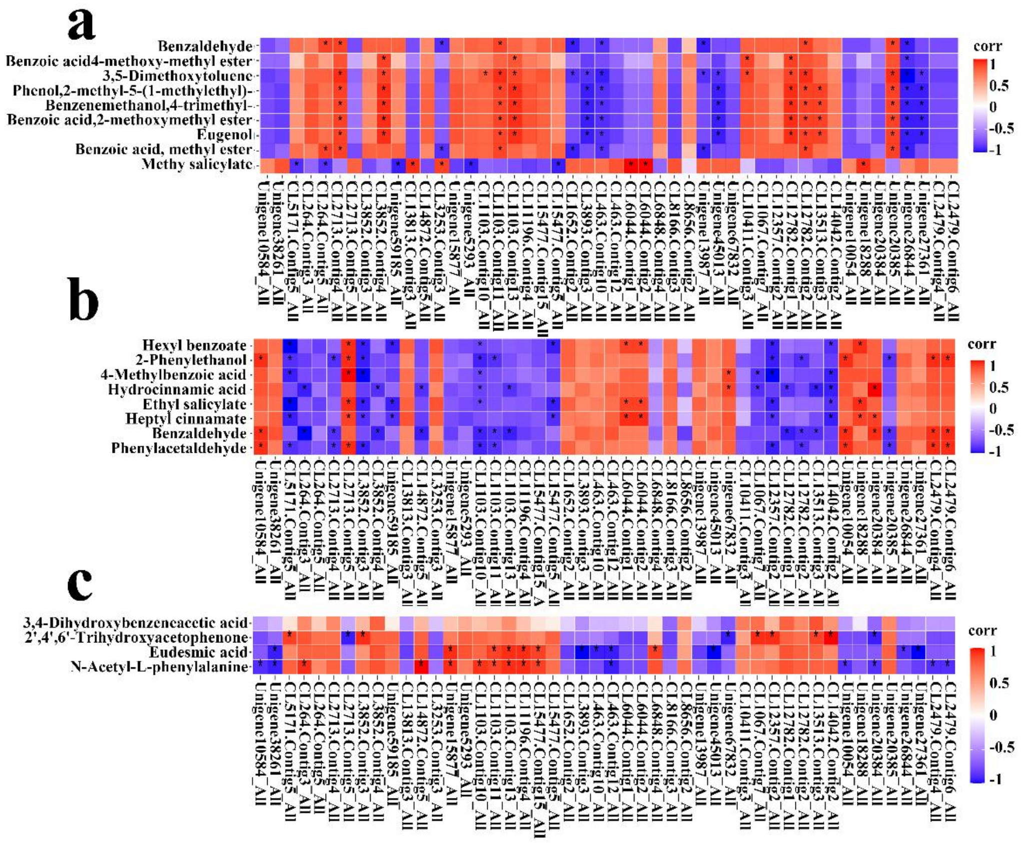

Figure. S25 Correlation analysis of YJ1 vs.YJ2 phenylpropanoid pathway candidate genes and benzene/phenylpropanoid substances. a. GC-TOFMS, b. LC-MS (POS), c. LC-MS (NEG).

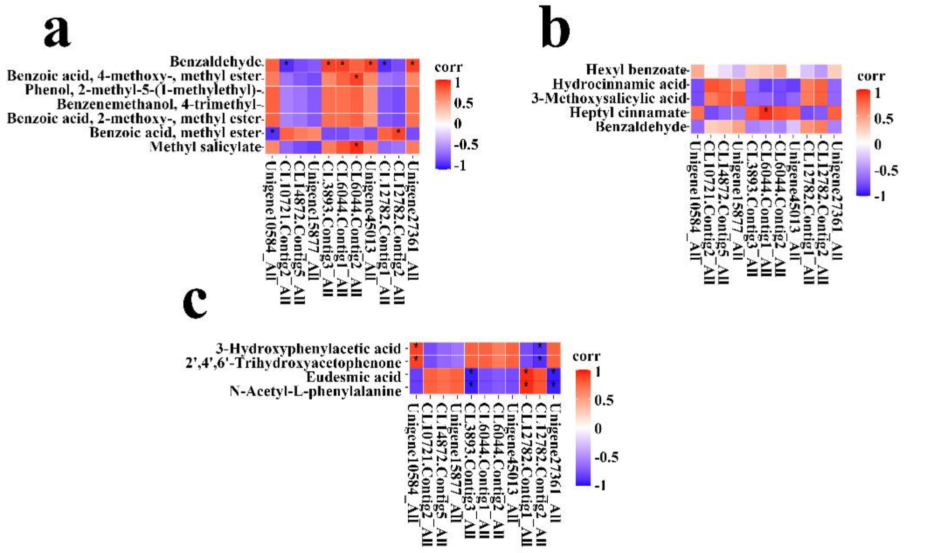

Figure. S26 Correlation analysis of YJ2 vs.YJ3 phenylpropanoid pathway candidate genes and benzene/phenylpropanoid substances. a. GC-TOFMS, b. LC-MS (POS), c. LC-MS (NEG).

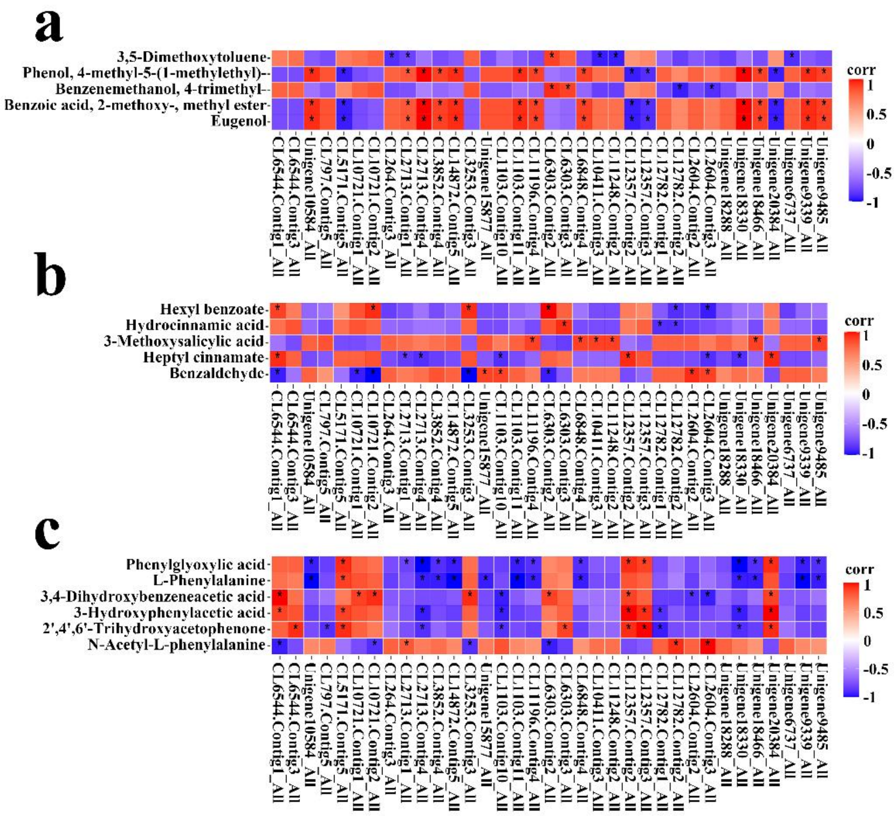

Figure. S27 Correlation analysis of YJ3 vs.YJ4 phenylpropanoid pathway candidate genes and benzene/phenylpropanoid substances. a. GC-TOFMS, b. LC-MS (POS), c. LC-MS (NEG).
