# Supplementary material for: Transcriptomic and metabolomic data reveal key genes that are involved in the phenylpropanoid pathway and regulate the floral fragrance of Rhododendron fortunei
Source: BMC Plant Biol. 2023 Jan 5;23:8. doi: 10.1186/s12870-022-04016-7 (PMC9814181; doi:10.1186/s12870-022-04016-7)
Supplement: Supplementary file 2 — Additional file 2: Table S1-S6. [file 12870_2022_4016_MOESM2_ESM.zip › Additional file/Table S4.docx]

| Number | substance name | Relative content(％) |
| --- | --- | --- |
| 1 | (+)-epi-Bicyclosesquiphellandrene | 27.20 |
| 2 | Benzoic acid, methyl ester | 16.41 |
| 3 | 2-Octanol | 12.19 |
| 4 | 3,5-Dimethoxytoluene | 3.94 |
| 5 | L-.alpha.-Terpineol | 8.79 |
| 6 | Methyl salicylate | 6.98 |
| 7 | 3-Carene | 4.97 |
| 8 | Linalool | 9.12 |
| 9 | Eucalyptol | 3.59 |
| 10 | 2-Nonenal, (E)- | 3.03 |
| 11 | (-)-.beta.-Bourbonene | 2.50 |
| 12 | Bicyclo[3.1.0]hexane, 4-methylene-1-(1-methylethyl)- | 2.41 |
| 13 | Naphthalene, 1,2,3,5,6,8a-hexahydro-4,7-dimethyl-1-(1-methylethyl)-, (1S-cis)- | 2.26 |
| 14 | 2-Hexenal, (E)- | 2.24 |
| 15 | 3a,7-Methano-3aH-cyclopentacyclooctene, 1,4,5,6,7,8,9,9a-octahydro-1,1,7-trimethyl-, [3aR-(3a.alpha.,7.alpha.,9a.beta.)]- | 2.08 |
| 16 | Benzene, 1,2-dimethoxy-4-(1-propenyl)- | 1.81 |
| 17 | 2,6-Nonadienal, (E,Z)- | 1.71 |
| 18 | Naphthalene, 1,2,3,4,4a,5,6,8a-octahydro-7-methyl-4-methylene-1-(1-methylethyl)-, (1.alpha.,4a.beta.,8a.alpha.)- | 1.61 |
| 19 | Spiro[5.5]undec-2-ene, 3,7,7-trimethyl-11-methylene-, (-)- | 0.95 |
| 20 | Benzoic acid, 2-methoxy-, methyl ester | 0.81 |
| 21 | .alfa.-Copaene | 0.68 |
| 22 | Bicyclosesquiphellandrene | 0.62 |
| 23 | 3-Cyclohexen-1-ol, 4-methyl-1-(1-methylethyl)-, (R)- | 0.59 |
| 24 | Hexanal | 0.59 |
| 25 | Methyl 6,6-dimethylbicyclo[3.1.1]hept-2-ene-2-carboxylate | 0.50 |
| 26 | Methyleugenol | 0.41 |
| 27 | Eugenol | 0.40 |
| 28 | Bicyclo[2.2.1]heptan-2-ol, 1,7,7-trimethyl-, acetate, (1S-endo)- | 0.38 |
| 29 | Cyclohexene, 4-ethenyl-4-methyl-3-(1-methylethenyl)-1-(1-methylethyl)-, (3R-trans)- | 0.29 |
| 30 | Phenol, 2-methoxy-4-(1-propenyl)-, acetate | 0.27 |

WT

pCAMBIA1302-SAMT

| Number | substance name | Relative content(％) |
| --- | --- | --- |
| 1 | Benzoic acid, methyl ester | 20.27 |
| 2 | Linalool | 12.28 |
| 3 | .alpha.-Terpineol | 10.58 |
| 4 | (+)-epi-Bicyclosesquiphellandrene | 6.47 |
| 5 | 3-Carene | 6.38 |
| 6 | 2-Octanol | 6.17 |
| 7 | 2-Nonenal, (E)- | 5.06 |
| 8 | Cyclohexene, 4-ethenyl-4-methyl-3-(1-methylethenyl)-1-(1-methylethyl)-, (3R-trans)- | 4.82 |
| 9 | Eucalyptol | 4.39 |
| 10 | 3,5-Dimethoxytoluene | 3.38 |
| 11 | 2-Hexenal, (E)- | 2.06 |
| 12 | 2,6-Nonadienal, (E,Z)- | 1.88 |
| 13 | (-)-.beta.-Bourbonene | 1.82 |
| 14 | Methyl salicylate | 1.18 |
| 15 | 1,5-Cyclodecadiene, 1,5-dimethyl-8-(1-methylethylidene)-, (E,E)- | 0.72 |
| 16 | .gamma.-Muurolene | 0.62 |
| 17 | Linalyl acetate | 0.42 |
| 18 | Naphthalene, 1,2,3,4,4a,5,6,8a-octahydro-7-methyl-4-methylene-1-(1-methylethyl)-, (1.alpha.,4a.beta.,8a.alpha.)- | 0.41 |
| 19 | Hexanal | 0.40 |
| 20 | Bicyclo[2.2.1]heptan-2-ol, 1,7,7-trimethyl-, acetate, (1S-endo)- | 0.37 |
| 21 | D-Limonene | 0.36 |
| 22 | cis-Muurola-4(15),5-diene | 0.34 |
| 23 | Benzene, 1,4-dimethyl-2-(2-methylpropyl)- | 0.34 |
| 24 | .gamma.-Elemene | 0.30 |
| 25 | Benzene, 1,2-dimethoxy-4-(1-propenyl)- | 0.28 |
| 26 | 1-Octen-3-ol | 0.28 |
| 27 | 3-Cyclohexen-1-ol, 4-methyl-1-(1-methylethyl)-, (R)- | 0.27 |
| 28 | Cyclohexanemethanol, .alpha.,.alpha.-dimethyl-4-methylene- | 0.25 |
| 29 | Phenol, 2-methoxy-4-(1-propenyl)-, acetate | 0.23 |
| 30 | .alpha.-Muurolene | 0.19 |

| 编号 | 名称 | 相对含量（％）Relative content |
| --- | --- | --- |
| 1 | Bicyclosesquiphellandrene | 24.83 |
| 2 | Benzoic acid, methyl ester | 14.11 |
| 3 | 3,5-Dimethoxytoluene | 12.46 |
| 4 | L-.alpha.-Terpineol | 10.98 |
| 5 | 2-Octanol | 9.68 |
| 6 | Methyl salicylate | 7.61 |
| 7 | 3-Carene | 3.87 |
| 8 | (-)-.beta.-Bourbonene | 3.46 |
| 9 | 2-Nonenal, (E)- | 3.04 |
| 10 | Naphthalene, 1,2,3,5,6,8a-hexahydro-4,7-dimethyl-1-(1-methylethyl)-, (1S-cis)- | 2.60 |
| 11 | Benzene, 1,2-dimethoxy-4-(1-propenyl)- | 2.53 |
| 12 | Naphthalene, 1,2,3,4,4a,5,6,8a-octahydro-7-methyl-4-methylene-1-(1-methylethyl)-, (1.alpha.,4a.beta.,8a.alpha.)- | 1.82 |
| 13 | Bicyclo[3.1.0]hexane, 4-methylene-1-(1-methylethyl)- | 1.73 |
| 14 | Caryophyllene | 1.53 |
| 15 | 2,6-Nonadienal, (E,Z)- | 1.52 |
| 16 | Benzoic acid, 2-methoxy-, methyl ester | 1.40 |
| 17 | (+)-epi-Bicyclosesquiphellandrene | 1.10 |
| 18 | Methyleugenol | 0.79 |
| 19 | 3-Cyclohexen-1-ol, 4-methyl-1-(1-methylethyl)-, (R)- | 0.62 |
| 20 | Cyclohexene, 4-ethenyl-4-methyl-3-(1-methylethenyl)-1-(1-methylethyl)-, (3R-trans)- | 0.61 |
| 21 | Eugenol | 0.58 |
| 22 | Hexanal | 0.41 |
| 23 | Bicyclo[2.2.1]heptan-2-ol, 1,7,7-trimethyl-, acetate, (1S-endo)- | 0.39 |
| 24 | .alpha.-Farnesene | 0.35 |
| 25 | 1,6,10-Dodecatrien-3-ol, 3,7,11-trimethyl-, (E)- | 0.29 |
| 26 | D-Alanine, N-(4-butylbenzoyl)-, heptyl ester | 0.27 |
| 27 | Benzenemethanol, .alpha.,.alpha.,4-trimethyl- | 0.23 |
| 28 | .gamma.-Terpinene | 0.16 |
| 29 | Phenol, 2-methoxy-4-(1-propenyl)- | 0.16 |
| 30 | .alpha.-Calacorene | 0.15 |

pCAMBIA1302
